# Ohmic Losses Dominated Electrode Fouling during Long-Term Aluminum Electrocoagulation of Hypersaline and Divalent Cation-Rich Oilfield-Produced Water

**DOI:** 10.1021/acsestengg.5c00628

**Published:** 2025-10-17

**Authors:** Sanket Joag, Jonathan Kiesewetter, Shankararaman Chellam

**Affiliations:** † Department of Civil & Environmental Engineering, 14736 Texas A&M University, College Station, Texas 77843-3136, United States; ‡ Process, Water & Heat Transfer Engineer, Global Production − Facilities Engineering, 3994 ConocoPhillips, 925 N. Eldridge Parkway, Houston, Texas 77079, United States; § Department of Chemical Engineering, Texas A&M University, College Station, Texas 77843-3122, United States

**Keywords:** wastewater treatment, electrochemistry, passivation, corrosion, Permian Basin, electrochemical impedance spectroscopy

## Abstract

Electrode behavior was elucidated during long-term galvanostatic electrocoagulation (aluminum anode and aluminum cathode) of a hypersaline oilfield produced water rich in divalent cations. Electrode potentials progressively increased (i.e., fouling) for most operational conditions due to surface accumulation of calcite and brucite. The interfacial resistance resulting from partial insulation by electrodeposited salts was quantified by using electrochemical impedance spectroscopy. The potential drop associated with this resistance correlated strongly and positively with the increased overpotential required to maintain the galvanostatic operation and was statistically indistinguishable from the calculated ohmic drop, confirming that electrode fouling could be fully attributed to ohmic effects. This also ruled out the occurrence of electrochemical side reactions at elevated potentials, despite their thermodynamic feasibility (note that *H*_2_(*g*) evolution is a non-Faradaic chemical reaction). We evaluated polarity reversal (PR) as a fouling mitigation strategy to restore electrode performance over a 4-fold variation in current density and a 100-fold variation in PR interval. The PR interval did not significantly influence performance, and fouling was effectively mitigated only at the highest applied current density (200 mA·cm^–2^). Results indicated the existence of a threshold current density and associated hydrogen bubble generation rate necessary to effectively control electrode fouling under the experimental conditions investigated. Foulant deposition also hindered the migration of electrodissolved aluminum ions away from the anode, facilitating their supersaturation, nucleation, precipitation, and entrapment, thereby decreasing the apparent Faradaic efficiency of coagulant dosing.

## Graphical abstract

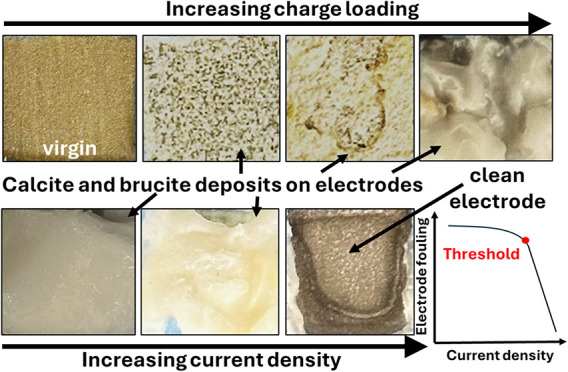

## 1 Introduction

Electrocoagulation, wherein coagulants are generated *in situ* by intentional corrosion of sacrificial electrode(s),^1–3^ offers several advantages over conventional coagulation because in addition to destabilizing suspended materials and organic matter, it can (i) remove dissolved solids via cathodic electrodeposition^4,5^ and floc-uptake,^3,6,7^ (ii) decrease lifecycle greenhouse gas emissions, (iii) reduce usage, storage, and transportation of hazardous chemicals, and (iv) oxidize contaminants via cathodic surface reactions.^8,9^ While these pathways have been recognized for some time in the electrochemistry/corrosion literature, their environmental applications are only recently being explored, leaving several unresolved barriers for wider implementation.^10,11^ Additionally, electrocoagulation units are modular and transportable, offering significant logistical advantages for low treatment capacities, particularly in remote areas or where treatment is only temporarily required.^5,10^ One such application is the treatment of produced water generated during oil and gas exploration and production, which in certain cases requires treatment only for limited periods spatially, often making the construction of large and permanent treatment infrastructure unnecessary and impractical.^12^

The Permian Basin is the world’s largest producer of unconventional shale oil cogenerating approximately a billion gallons of hypersaline produced water daily^13^ with total dissolved solids (TDS) concentration often exceeding 100,000 mg·L^–1^.^12,13^ Such large volumes provide opportunities for internal reuse^14^ and beneficial reuse^13,15^ for which electrocoagulation might be uniquely suited because the high electrical conductivity of produced water reduces ohmic overpotentials thereby lowering energy costs. However, this comes with challenges such as (i) multifold exacerbation of anodic corrosion due to high chloride ion levels,^16,17^ (ii) need for reoxygenation if electrocoagulation is integrated with downstream aerobic biological processes, because the inherently low dissolved oxygen in high-salinity waters^18^ is further depleted by cathodic reduction,^2^ and (iii) cathode fouling arising from high concentrations of divalent cations.^1^ Treatment and reuse of this wastewater has gained recent attention as a mechanism to reduce the volume sent to saltwater disposal, coupled with corporate environmental, social, and governance policies.^19,20^ Nevertheless, electrocoagulation performance and implementation in the Permian Basin (and many other locations) remain largely unresolved due to the produced water’s complex composition (including transient variations) and high levels of suspended and dissolved components.^10,15,21^

The overarching goals of this work were to mechanistically investigate electrode fouling and evaluate periodic polarity reversal (PR) as an approach for fouling mitigation during electrocoagulation of Permian Basin produced water. PR involves switching the supplied current direction at regular intervals, thereby changing the electrode polarity (i.e., anode ⇌ cathode). To date, PR has been exclusively studied for soft, freshwater matrices^22,23^ with contrasting findings (SI Table S3) typically inferred from relatively short-term process-level assessments that are sometimes accompanied by detailed aqueous chemistry and solid(s) characterization^22–25^ but often omitting detailed electrochemistry principles. Herein, we elucidate electrochemical and interfacial phenomena associated with PR in produced water having ≳30-fold higher concentrations of divalent cations than earlier reports, along with associated responses of the fouling layer. PR can alleviate performance degradation arising from both anode passivation and cathode fouling. In the former case, PR disrupts the buildup of insulating oxide films upon periodic reversal to cathodic polarity by facilitating hydrogen evolution and physical film disruption.^23,24^ In the latter case, PR decreases precipitation and deposition of scale-forming compounds such as calcium carbonate or magnesium hydroxide upon periodic reversal to anodic polarity by avoiding pH increase in the cathode microenvironment.^26,27^ We specifically target cathodic fouling because anodic passivation is insignificant in high chloride environments.^16,17^ The hypothesized fouling control mechanisms by PR under typical operating conditions include: (i) physical dislodgement of cathodic foulants by electrochemically generated H_2_ gas,^1,22,24^ (ii) removal of anodic surface deposits by electrodissolving the underlying electrode material,^1,22^ (iii) dissolution, weakening adhesion, and eventual detachment of cathodic foulants via decreasing pH in its microenvironment when it is reversed to anode,^1,22,27^ and (iv) hydrodynamically shearing the fouling layer in continuous-flow reactors.^22,24^

Primary objectives were to (i) evaluate the long-term performance of aluminum anode–aluminum cathode electrocoagulation with PR, (ii) establish the dominant role of divalent cations on corrosion behavior, and (iii) investigate electrode fouling and mitigation mechanisms. Electrochemical techniques such as open-circuit potential (OCP) and potentiodynamic polarization were used to determine the effects of dissolved constituents on aluminum dissolution. Ohmic overpotential was quantified by using electrochemical impedance spectroscopy (EIS). (Electro)­deposits and flocs were comprehensively characterized using X-ray photoelectron spectroscopy (XPS), X-ray diffraction (XRD), and Fourier Transform Infrared (FTIR) spectroscopy.

## 2 Materials and Methods

### 2.1 Produced Water

Samples were taken from producing wells in the Delaware Basin in west Texas and southern New Mexico whose composition (SI Table S1) agreed with that reported for the broader Permian Basin.^28^ In short, they were slightly acidic (pH 6.4 ± 0.1) and hypersaline (TDS 120,000 ± 3200 mg·L^–1^) with high concentrations of iron (22 ± 2 mg·L^–1^), silicon (35 ± 2 mg·L^–1^ as SiO_2_), chloride (78,000 ± 2000 mg·L^–1^), sulfate (180 ± 5 mg·L^–1^), and divalent cations (calcium 3,530 ± 127 mg·L^–1^, magnesium 790 ± 22 mg·L^–1^, strontium 1048 ± 13 mg·L^–1^, and barium 6 ± 1 mg·L^–1^) resulting in total hardness of 13,250 ± 330 mg·L^–1^ as CaCO_3_. It was also turbid (85 ± 3 NTU) and laden with organic compounds (dissolved organic carbon, DOC 52.4 ± 1.2 mg·L^–1^).

### 2.2 Experiments

Electrocoagulation was performed with aluminum plate electrodes (99.28% purity, SI Table S2) in a 250 mL borosilicate batch reactor with a 100 mL working volume without adjusting the pH (initial pH of 6.4 ± 0.1). The pH dropped in all electrocoagulation experiments, the magnitude of which increased with charge loading (CL, charge supplied per unit volume) and current density (CD, current passed per unit electrode surface area), as summarized in SI Figure S11, but is not discussed in detail because we have reported these trends recently.^5^ The exposed electrode area was precisely set to 2 cm^2^ by masking the remaining surface with nonconductive tape. Experiments were conducted in batch mode to reduce the influence of hydrodynamic shear, allowing us to largely isolate electrochemical principles underlying fouling. Electrodes were spaced 2 cm apart, and a custom-fabricated lid was used to secure all components (SI Figure S1). The total CL was kept constant at 32,160 C·L^–1^ in all cases, resulting in experimental durations of 2–9 h based on the CD. We emphasize that this constituted a greater than 15-fold higher CL than previous PR investigations, thereby generating substantially longer-term data than what are currently available.^29–32^ Galvanostatic experiments were conducted at CDs of 50, 100, and 200 mA·cm^–2^ with PR intervals of 5.36, 53.6, and 536 s and a control condition without PR. PR was implemented “symmetrically” such that each electrode alternated roles, functioning as the anode for half the duration and as the cathode for the remaining half. All potentials were measured and reported with respect to a saturated Ag/AgCl reference electrode (*E*_Ag/AgCl_ = 0.197 V versus SHE).^17^ To ensure consistent surface conditions, electrodes were polished with 2000-grit silicon carbide sandpaper, rinsed thoroughly with ultrapure water, degreased with acetone, and flushed with N_2(g)_ before each experiment.^33^ A potentiostat (1010E, Gamry Instruments) was utilized either in galvanostatic mode for providing constant current throughout the experimental duration (no PR) or cycled in chronopotentiometry mode to intermittently change the current direction (for PR). EIS was performed intermittently to measure ohmic overpotentials (SI Section S4). All post-processing was performed using Gamry Echem Analyst software.

### 2.3 Characterization of Electrodes, Flocs, and Electrodeposited Solids

The morphology and elemental composition of electrodes (pre- and post-electrocoagulation) and flocs were analyzed using scanning electron microscopy and energy-dispersive X-ray spectroscopy (SEM-EDS, Tescan Vega 3 SEM with Oxford EDS). Major functional groups and chemical states were identified via FTIR (Thermo Scientific Nicolet iS10) and XPS (PHI 5700, Physical Electronics), respectively. Polymorphs of electrodeposited solids were examined via powder XRD (AXS D8 Davinci, Bruker Instruments) after rinsing multiple times with ultrapure water to reduce otherwise extraordinarily dominant halite peaks.^12,34,35^ SI Section S4 provides additional details.

## 3 Results and Discussion

### 3.1 Aluminum Electrodes Behaved Similarly in Produced Water and a Synthetic Electrolyte with Only Monovalent Ions

Aluminum leaching was explored for 9 h without applying an electrical potential using (i) Permian Basin produced water and (ii) synthetic solution lacking divalent cations and matching conductivity (130 mS·cm^–1^ by adding NaCl) and alkalinity of the produced water (65 ± 5 mg·L^–1^ as CaCO_3_ by adding NaHCO_3_) at pH 6.4 ± 0.1 termed “monovalent electrolyte” (Figure 1a). Chemically leached concentrations reached 2 ± 0.2 mg·L^–1^·cm^–2^ in both cases, demonstrating similar aluminum corrosion in the absence of current passage. Continuous aluminum release purely by chemical corrosion over the entire duration suggested that passivation was effectively suppressed, similar to our recent short-term experiments.^5^ Despite the presence of electron-donating moieties like hydrophobic organics and sulfates in the produced water, which reduce chloride-induced corrosion by adsorbing strongly on electrodes,^36–38^ leached aluminum concentrations were statistically similar (α = 0.05) for the two electrolytes, suggesting minimal influence of non-chloride anions and organic moieties.^5^ This can be attributed to their significantly lower relative concentrations with respect to chloride ( $1100\;\frac{{Cl}^{-}}{{SO}_{4}^{2-}}$ and $440\;\frac{{\mathrm{Cl}}^{-}}{\mathrm{DOC}}\;\mathrm{mol}/\mathrm{mol}$ ), aligning with our recent results that identified chloride as the dominant driver of aluminum dissolution.^5^ OCPs were statistically indistinguishable for produced water and the monovalent electrolyte and were recorded at −749 ± 10 mV, corroborating the minimal influence of non-chloride anions and organics (Figure 1b). This OCP was more negative than what was measured for another Permian Basin sample with slightly lower chloride ion concentrations,^5^ further highlighting the influence of high chloride concentrations on OCP.^39^ These OCP values are higher than the theoretical potential for aluminum dissolution (SI Reaction R1, −1857 mV) but lower than those for other common anodic reactions like water oxidation (oxygen evolution reaction, OER) and chloride oxidation reaction (SI Reactions R2 and R3) that have theoretical potentials of +1032 mV and +1161 mV, respectively, confirming aluminum oxidation as the dominant anodic reaction per mixed potential theory.^40^ Conversely, the values of the OCP remained lower than the corresponding theoretical potentials for hydrogen evolution and oxygen reduction (SI Reactions R4 and R5) that have theoretical potentials of −574.6 mV and +581 mV (at pH 6.4), respectively, suggesting that both these reactions are feasible on cathodic sites of electrodes.

**Figure 1:**
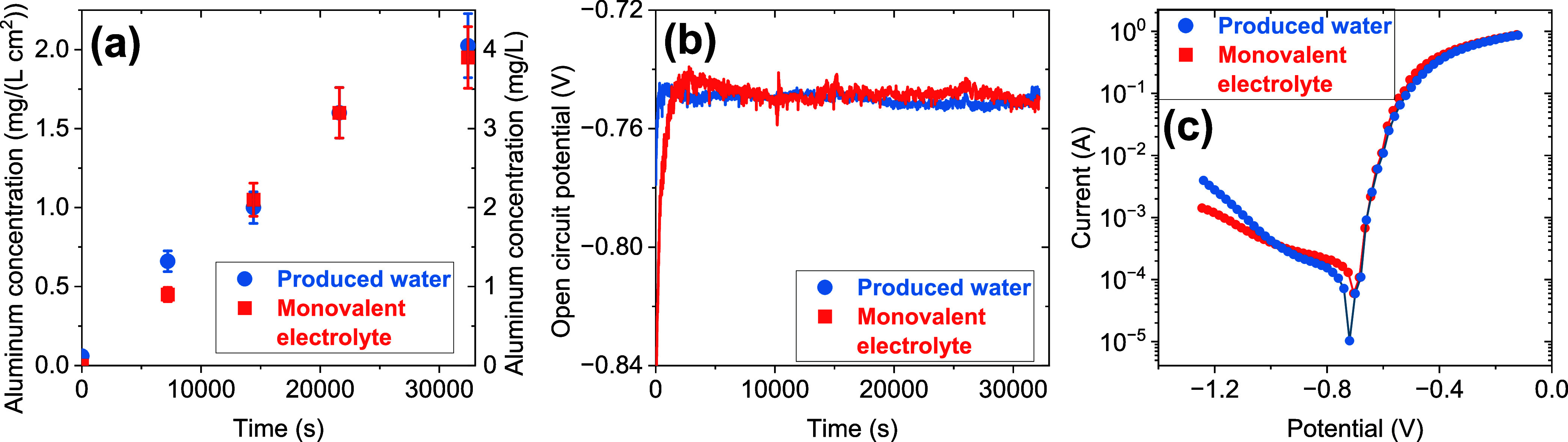
(a) Temporal evolution of chemically leached aluminum concentrations in the bulk solution normalized to electrode surface area (left ordinate, mg·L^–1^·cm^–2^) and without normalization (right ordinate, mg·L^–1^) when no electrical potential was applied. (a) Experiments conducted with Permian Basin produced water and a monovalent ion electrolyte (conductivity matched to 130 mS·cm^–1^ and alkalinity matched to 65 mg·L^–1^ as CaCO_3_), (b) temporal profiles of the electrode open-circuit potential (OCP) in both electrolytes, and (c) semilog potentiodynamic polarization curves, plotted as current (A) in log-scale versus applied potential (V) for both electrolytes.

Potentiodynamic polarization measurements conducted to quantify corrosion kinetics (Figure 1c) revealed corrosion potentials (−745 ± 20 mV) that were statistically indistinguishable from open-circuit values. Tafel extrapolation yielded similar exchange CDs of 178 ± 10 and 193 ± 4 μA·cm^–2^ for produced water and monovalent electrolyte, respectively, corresponding to corrosion rates of 0.49 ± 0.02 and 0.53 ± 0.01 mm·year^–1^, respectively. The statistically similar (α = 0.05) CDs and corrosion rates further validated the use of NaCl in control experiments to isolate the effects of the produced water matrix (including divalent cations) on electrochemical behavior.

### 3.2 Increased Potentials during Long-Term Electrocoagulation with Polarity Reversal were Directly Attributed to Ohmic Losses Arising from Surface (Electro)­deposition on Electrodes

#### 3.2.1 Constant Anodic Potentials in the Synthetic Monovalent Ion Electrolyte and Experiments for Both Electrolytes without PR

Upon application of current, the anode exhibited a positive overpotential over the cathode. All monovalent electrolyte experiments exhibited a constant anodic potential (*E*_anode_) throughout the entire CL (red dots in Figure 2a–l). Experiments without PR in produced water (Figure 2a,e,i) closely mirrored aluminum dissolution trends in the monovalent electrolyte, further reinforcing statistically indistinguishable (α = 0.05) corrosion kinetics for both electrolytes in the absence of PR. The anodic potential at the experimental onset (t = 0 s) was directly proportional to CD (slope = 3.29 mV mA^–1^·cm^2^), referring to the increased activation overpotential^17,36^ (SI Section S5). At 50 mA·cm^–2^, the initial *E*_anode_ was −580 ± 20 mV, corresponding to an overpotential (η_anode_ = *E*_anode_ – E_OCP_) of 170 ± 24 mV (Figure 2a–d). Doubling the CD to 100 mA·cm^–2^ more than doubled the η_anode_ to 390 ± 20 mV (*E*_anode_ – 360 ± 10 mV; Figure 2e–h). Quadrupling the CD to 200 mA·cm^–2^ nearly quadrupled the η_anode_ to 630 mV (*E*_anode_ – 121 ± 26 mV; Figure 2i–l). The magnitude of initial *E*_anode_ for all CDs in both the electrolytes was more positive than the theoretical potential for aluminum dissolution, and remained well below the theoretical potentials for competing anodic reactions such as oxygen evolution and chloride oxidation, confirming that aluminum dissolution was the dominant anodic reaction (SI section S6).^5^ Moreover, insignificant anodic passivation was inferred from the constant magnitude of *E*_anode_ (−590 ± 30 mV for 50 mA·cm^–2^, −370 ± 20 mV for 100 mA·cm^–2^, and −130 ± 30 mV for 200 mA·cm^–2^) throughout the range of CLs tested with the monovalent electrolyte, irrespective of CD or PR interval. Moreover, a constant current at increasing potential forming a plateau in the log I–V plot, denoting active-to-passive transition, was absent.^36^ High corrosion current for these potentials and the absence of a distinct plateau corresponding to passivation in the potentiodynamic polarization corroborated this observation (Figure 1c). This is consistent with aluminum behavior in high-chloride environments, where Cl^–^ ions adsorb onto the oxide layer, penetrate defect sites, and completely rupture the passive film,^41,42^ suppressing the buildup of passivation overpotential typically observed in low-chloride systems, where the compact oxide film progressively increases interfacial resistance.^43–45^

**Figure 2:**
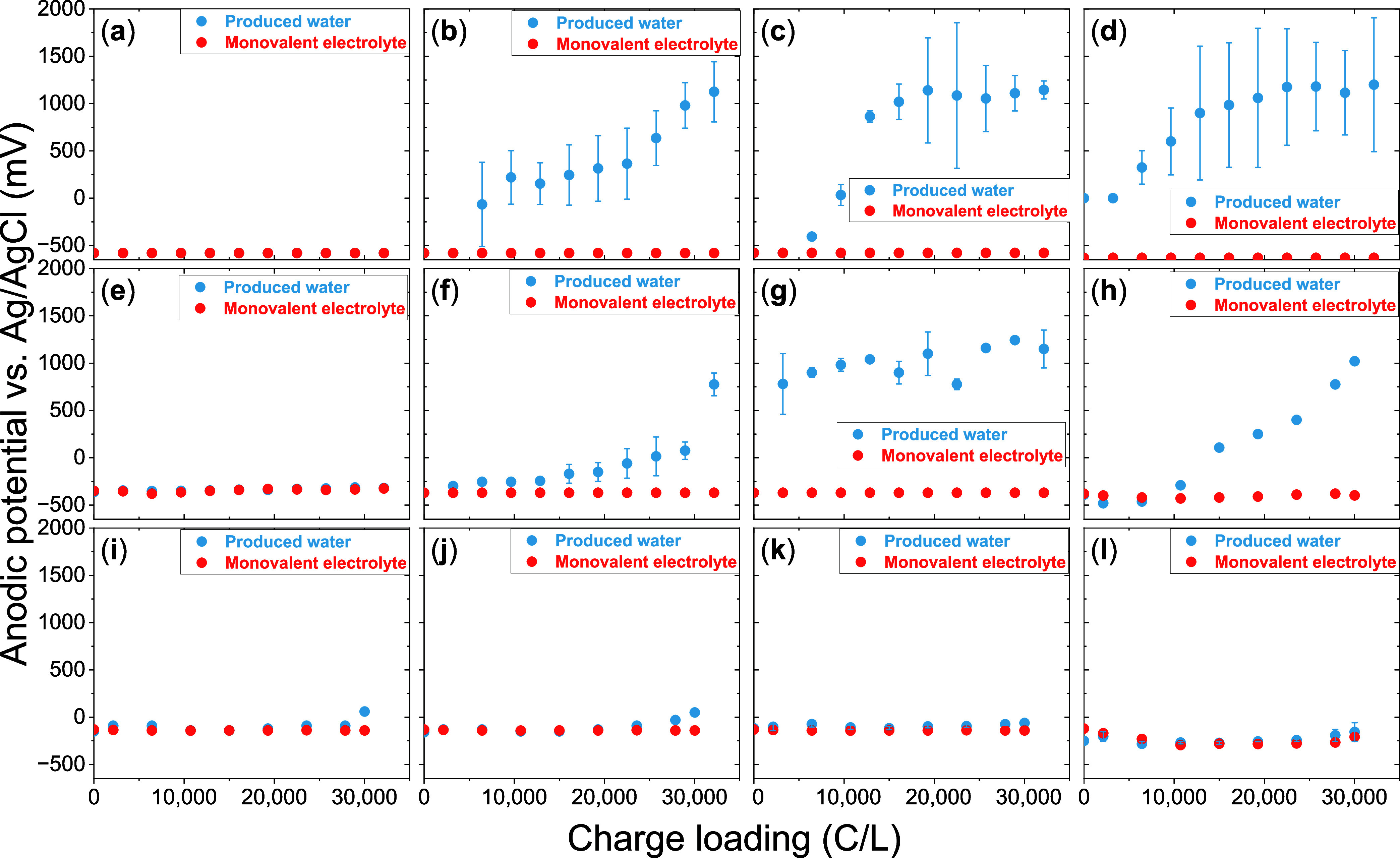
Anodic potentials for CD = 50 mA·cm^–2^ with (a) no PR, (b) PR interval = 5.36 s, (c) PR interval = 53.6 s, (d) PR interval = 536 s, CD = 100 mA·cm^–2^ with (e) no PR, (f) PR interval = 5.36 s, (g) PR interval = 53.6 s, (h) PR interval = 536 s, CD = 200 mA·cm^–2^ with (i) no PR, (j) PR interval = 5.36 s, (k) PR interval = 53.6 s, (l) PR interval = 536 s. Red dots indicate the potentials in the synthetic monovalent ion electrolyte, while blue dots indicate potentials in the produced water. In parts (a) and (e), the blue symbols are covered by the overlaid red markers and are therefore not visible.

#### 3.2.2 Anodic Potentials Increased in Magnitude for Produced Water Electrocoagulation with PR

In contrast to the synthetic monovalent electrolyte, *E*_anode_ progressively increased in experiments with produced water involving PR over CL for CDs of 50 and 100 mA·cm^–2^, irrespective of PR interval (blue dots in Figure 2b–d,f–h,j–l). This rise is unlikely to have resulted from an increase in activation overpotential because conditions were unchanged compared to experiments without PR and controls, where potential remained constant. Passivation overpotential can also be excluded as a possibility, given the high chloride concentration (detailed in Section 3.2.1).^17^ Furthermore, negligible concentration polarization was expected since anodic dissolution ensured a continuous release of Al^3+^ ions at the electrode interface, minimizing depletion of reactive species near the surface.^1,46^ Having systematically and rigorously ruled out other possible contributors, we attributed the observed increase in *E*_anode_ primarily to growing ohmic overpotentials, likely resulting from the accumulation of resistive surface deposits that impeded ionic transport at the electrode–electrolyte interface (SI section S5).

This inference was first qualitatively supported by visually inspecting the electrodes post-electrocoagulation (after CL of 32,160 C·L^–1^, SI Figure S2). Without PR, the anode displayed significant corrosion and visible material loss (SI Figure S2a,e,i), and the cathode was covered entirely with white precipitates. In PR experiments, particularly with CD = 50 and 100 mA·cm^–2^, both electrodes were covered with substantial white deposits (detailed in Section 3.4) to a similar extent. This was expected as electrodes alternated as the anode and cathode for half the total time (symmetric PR).

#### 3.2.3 Increased Magnitude of Cathodic Potentials during Experiments with and without PR

Cathodic potentials (*E*_cathode_) remained constant throughout the duration of each synthetic monovalent ion electrolyte experiment (red dots in Figure 3a–l). *E*_cathode_ became more negative (from −1800 to −2000 and to −2250 mV) as CD increased (from 50 to 100 and to 200 mA·cm^–2^) due to the higher magnitude of activation overpotential. Note that a more negative potential relative to the equilibrium potential (OCP) signifies a higher cathodic overpotential (η_cathode_). In contrast, for produced water electrocoagulation without PR, the *E*_cathode_ became increasingly negative as CL increased over the experimental duration (Figure 3a,e,i), indicating a rise in η_cathode_. This behavior is attributed to persistent fouling on the cathode surface, which remained cathodic over the entire experimental duration (because polarity was not reversed). For example, at 50 mA·cm^–2^, the final *E*_cathode_ in the no-PR case reached −3300 ± 200 mV, whereas in the monovalent electrolyte, it remained constant at −1770 ± 30 mV. When PR was incorporated at 50 mA·cm^–2^, the *E*_cathode_ reached −2,811 ± 25 mV. The greater change in the *E*_cathode_ in the case of no PR was attributed to the cathode remaining cathodic for the entire duration, whereas the same was true only for half the duration with symmetric PR. Similarly, at 100 mA·cm^–2^, the *E*_cathode_ was −3980 ± 70 mV (no PR) and −3608 ± 472 mV (with PR), respectively, compared to the potential in the monovalent electrolyte (−2020 ± 43 mV). In contrast, at the highest CD investigated (200 mA·cm^–2^), *E*_cathode_ remained stable (at −2474 ± 88 mV) across all experiments with and without PR for both electrolytes, suggesting minimal fouling. Hence, we hypothesized that the extent of potential increase (both anodic and cathodic) compared to the initial potential correlated with fouling^47^ (compare SI Figure S2c,d,g,h with SI Figure S2k,l). This provided a plausible explanation for the relatively low potential increase observed at the highest CD and was investigated next.

**Figure 3:**
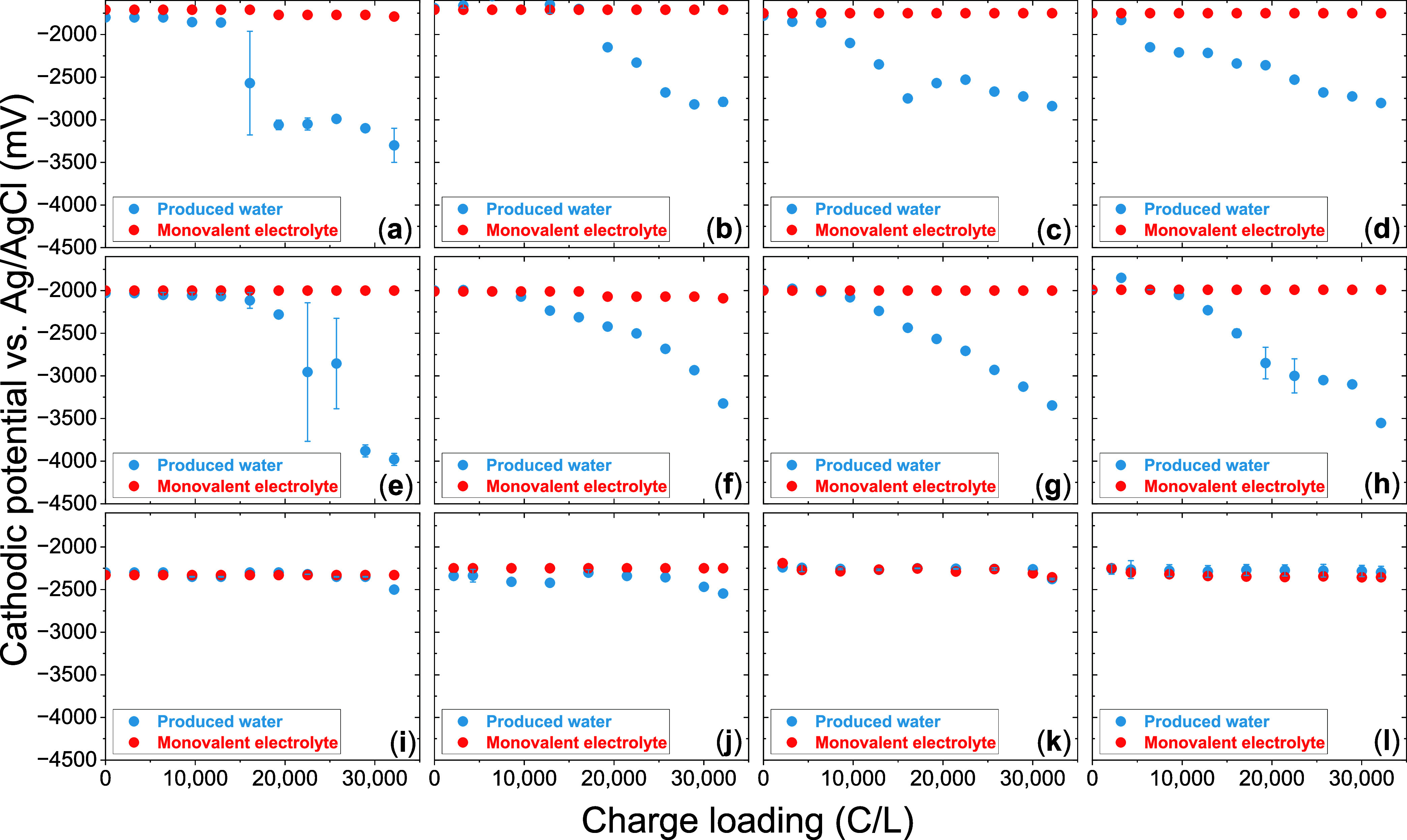
Cathodic potentials for CD = 50 mA·cm^–2^ with (a) no PR, (b) PR interval = 5.36 s, (c) PR interval = 53.6 s, (d) PR interval = 536 s, CD = 100 mA·cm^–2^ with (e) no PR, (f) PR interval = 5.36 s, (g) PR interval = 53.6 s, (h) PR interval = 536 s, CD = 200 mA·cm^–2^ with (i) no PR, (j) PR interval = 5.36 s, (k) PR interval = 53.6 s, (l) PR interval = 536 s. Red dots indicate the potentials in the synthetic monovalent ion electrolyte, while blue dots indicate potentials in the produced water.

### 3.3 EIS Revealed Ohmic Contributions were Predominantly Responsible for Increased Anodic Overpotentials

#### 3.3.1 Surface Deposits Increased Interfacial Resistance by Reducing Electrode–Electrolyte Contact

EIS can distinguish between different interfacial processes based on their individual time constants.^48,49^ Therefore, it was employed to monitor the time-evolution of ohmic resistance and to elucidate mechanisms responsible for potential increase during electrocoagulation by modeling its response via constructing equivalent circuits comprising idealized elements.^50^ Each subfigure in Figure 4 represents Nyquist plots for produced water at successive CLs along an experiment, each with a total CL of 32,160 C·L^–1^. SI Figure S4 displays the same data in three dimensions, incorporating CL on the *z*-axis to visualize the progressive increase in interfacial resistance with increasing CL.

**Figure 4:**
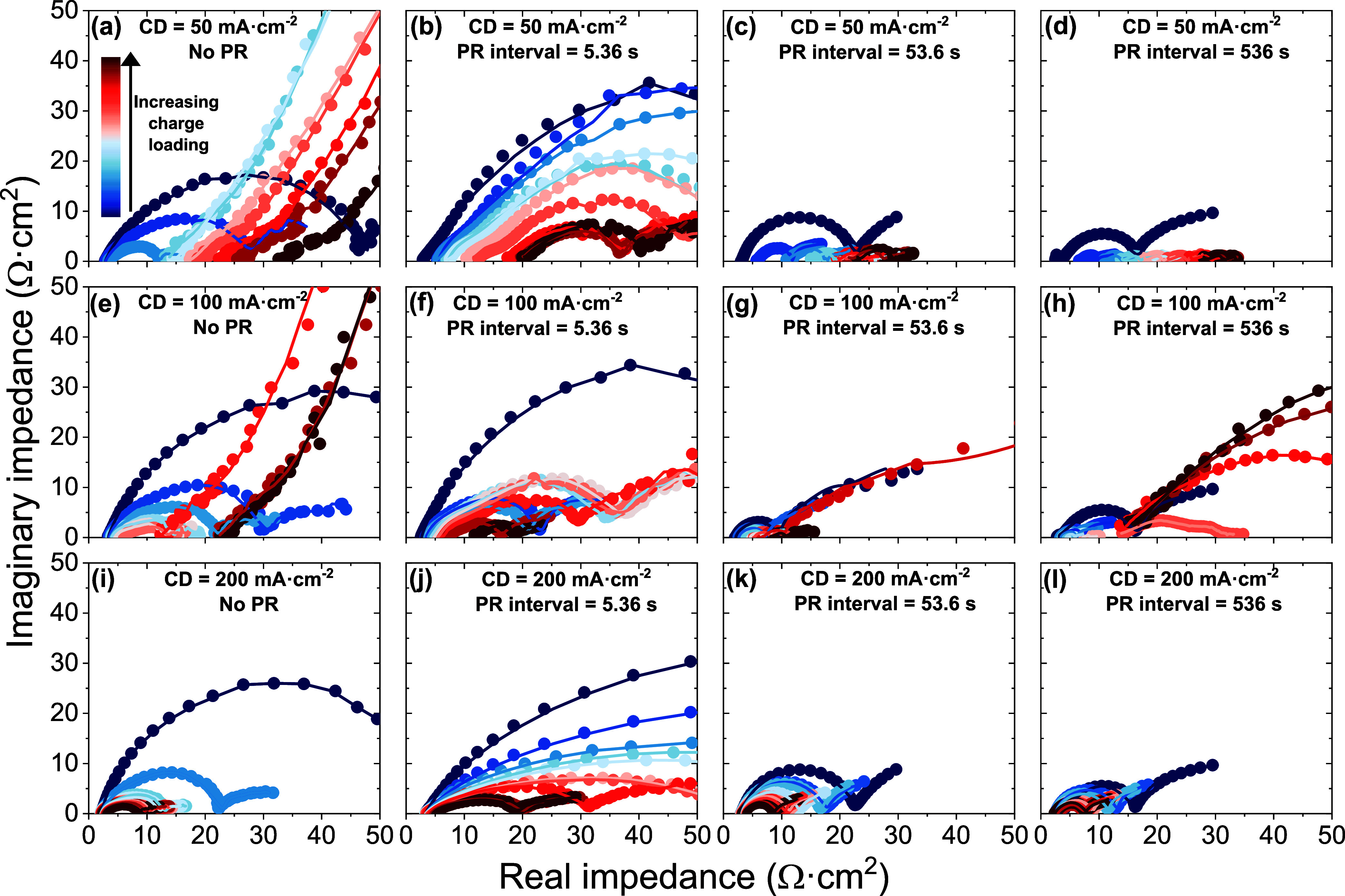
Nyquist plots for (a) CD = 50 mA·cm^–2^, no PR, (b) CD = 50 mA·cm^–2^, PR interval = 5.36 s, (c) CD = 50 mA·cm^–2^, PR interval = 53.6 s, (d) CD = 50 mA·cm^–2^, PR interval = 536 s, (e) CD = 100 mA·cm^–2^, no PR, (f) CD = 100 mA·cm^–2^, PR interval = 5.36 s, (g) CD = 100 mA·cm^–2^, PR interval = 53.6 s, (h) CD = 100 mA·cm^–2^, PR interval = 536 s, (i) CD = 200 mA·cm^–2^, no PR, (j) CD = 200 mA·cm^–2^, PR interval = 5.36 s, (k) CD = 200 mA·cm^–2^, PR interval = 53.6 s, (l) CD = 200 mA·cm^–2^, PR interval = 536 s. CL increases as we go from blue curves to red curves. Experimental data points are denoted by dots, where the continuous curves depict the modeled circuit. Nyquist plots in parts a, e, and (i) are constructed for the cathode (no PR).

For no PR, EIS was performed only on the cathode (Figure 4a,e,i), given insignificant anodic passivation. Because only symmetric PR was considered, EIS was performed on a single electrode since both would have fouled to a similar extent (being equivalently cathodic and anodic for half the total duration). In these plots, the *x*-intercept (real resistance) at high frequencies (i.e., leftmost intercept) corresponded to the uncompensated ohmic resistance (*R*_s_), which encompassed both the bulk electrolyte resistance and any resistance associated with restricted ionic mobility and/or longer ionic conducting path due to surface fouling or reduced effective electrode area^47,51,52^ (SI Figure S2). The electrical conductivity of the electrolyte remained invariant at 130 mS·cm^–1^, validating that its bulk ohmic resistance remained effectively constant across all experiments (SI Section S5). Consequently, any observed increase in *R*_s_ during successive impedance measurements was primarily attributed to changes in resistance offered at the electrode surface, likely due to the accumulation of insulating foulant deposits.^47,53^ The calculated theoretical resistance based solely on bulk conductivity and cell geometry was 2.7 ± 0.2 Ω·cm^2^ (SI Section S5), which corresponded well with the x-intercept measured in the first cycle of EIS spectra for most experimental conditions (2.64 ± 0.56 Ω·cm^2^) (Figure 4). It should be noted that this first cycle did not represent a true pre-electrocoagulation baseline measurement since aluminum electrodes contain an oxide film.^17,54^ This passive layer broke down during the initial stages of electrolysis, lowering both ohmic and charge transfer resistances. Therefore, EIS measurements just prior to the commencement of electrocoagulation may overestimate the true ohmic resistance due to additional contributions from the passivation layer. To establish a consistent datum for tracking changes in *R*_s_, EIS measurements performed after a modest CL of 215 C·L^–1^ were considered the effective starting point for analysis. At this stage, fouling was minimal (SI Figure S3), allowing subsequent increases in *R*_s_ to be attributed primarily to the formation of surface deposits.

However, as electrocoagulation progressed (i.e., CL and time progressively increased), the real-axis intercept systematically increased (Figure 4a–h) for the two lowest CDs investigated. Specifically for 50 mA·cm^–2^, it increased by an order of magnitude (20.66 ± 5.33 to 37.0 ± 0.82 Ω·cm^2^) compared to an initial intercept of 2.61 ± 0.12 Ω·cm^2^. Similarly for 100 mA·cm^–2^, the intercept ranged between 11.96 ± 0.41 and 23.2 ± 0.25 Ω·cm^2^ from an initial intercept of 2.80 ± 0.54 Ω·cm^2^. These shifts demonstrated the existence of an additional interfacial resistance arising from surface deposition, which progressively increased over time due to deposition and accumulation of mineral precipitates (Section 3.4) acting as resistive layers.^47,55^ Moreover, this indicated that heterogeneously nucleated precipitates persisted on electrodes and substantially reduced the effective electrode–electrolyte contact,^47^ which has important mechanistic implications. Specifically, it challenges the commonly proposed hypothesis that anodic dissolution of the underlying metal, facilitated by PR, promoted the dislodgement of surface deposits.^1,22,24,56^ If the deposits themselves significantly hindered ionic transport by obstructing the electrode–electrolyte interface and increasing ohmic resistance, then the underlying aluminum substrate was no longer electrochemically accessible in fouled regions, rendering localized corrosion-driven detachment ineffective. This is a key novel contribution because no alternative electrochemical pathway could account for fouling mitigation on an electrochemically inaccessible electrode surface, leaving mechanical dislodgement, primarily via hydrogen bubble evolution, as the dominant mechanism. Furthermore, the formation of an acidic boundary layer during anodic polarization, which has been suggested as a secondary mechanism to promote deposit dissolution,^1,26^ did not appear to fully account for foulant persistence. The presence of substantial residual deposits on electrode surfaces even after prolonged operation suggested that any aluminum dissolution occurring during the anodic half-cycle was insufficient to mitigate fouling. This interpretation aligned with the consistently elevated values of interfacial resistance measured via EIS. In contrast, the resistance increased negligibly (<2 Ω·cm^2^) in all experiments (with and without PR) at 200 mA·cm^–2^ due to minimal accumulation of mineral precipitates.

Notably, the magnitude of *R*_s_ increase mirrored the rising trend of anodic potential: the largest increases were measured for the two lowest CDs, whereas both *R*_s_ and potential remained constant at the highest CD. This further reinforced the hypothesis that physical disruption of the surface deposit by hydrogen bubbles, which were released at the highest rate at 200 mA·cm^–2^, was the dominant fouling mitigation mechanism rather than electrochemical or chemical dissolution. This observation, along with the potential measurements (Figures 2 and 3), indicated the existence of a threshold CD (between 100 and 200 mA·cm^–2^) above which the release rate of hydrogen gas bubbles was sufficiently high to scour (electro)­deposits and the corresponding resistive built-up. Below the threshold, foulant accumulation and potentials were relatively similar at the two lower CDs investigated (50 and 100 mA·cm^–2^), suggesting that the hydrogen bubble generation rate was insufficient to mitigate fouling. The agreement between the electrochemical impedance and measured potentials further corroborated the hypothesis that surface fouling was the dominant contributor to the observed performance deterioration at low CDs. Comparing this value to the total measured overpotential increase enabled assessing the extent to which solid-phase buildup alone accounted for the observed potential rise, which is pursued in Section 3.3.2.

EIS spectra collected at multiple time points during each experiment were fitted using an equivalent circuit model (SI Tables S3–S14 summarize corresponding parameters). Across all conditions, Nyquist plots depicted two distinct depressed semicircular arcs, corresponding to the series assembly of two RQ circuits, one corresponding to a film of the nonconductive mineral deposits at the interface of the electrolyte and the other at the electrode–foulant film interface. Both semicircles were depressed, representing a constant phase element (*Q*) instead of a capacitor (*C*). At 50 and 100 mA·cm^–2^ (Figure 4a–h), these plots often deviated from even depressed semicircular shapes, appearing irregular and distorted (e.g., higher CLs in Figure 4c,d,g,h), likely due to nonuniform foulant deposition and resulting spatial variations in current distribution across the electrode surface. These distortions notwithstanding, the extracted *R*_s_ consistently increased, corroborating the visual evidence of progressive surface fouling and associated reductions in the effective electroactive area (SI Figure S3). Nyquist plots for 200 mA·cm^–2^ (Figure 4i–l) were more uniform in shape due to minimal surface fouling.

In contrast to increasing interfacial resistances, the charge transfer resistance (*R*_ct_), which indicates hindrance to the kinetics of electron transfer (Faradaic) reactions,^57^ generally decreased over the course of experimentation relative to established baseline measurements consistent with localized pitting corrosion and active electrode dissolution.^43,54^ This trend stands out at higher PR intervals (i.e., 53.6 and 536 s), where semicircle diameters are notably smaller. Applying current for extended periods without interruption (i.e., longer intervals between PR) promoted more extensive pitting,^58,59^ inducing the formation of a greater number of pits, subsequently increasing surface activation and decreasing charge transfer resistance. Fouling partially blocked electrode surfaces, thereby heterogeneously distributing the applied current and consequently increasing the local CD in the exposed regions. Such localized intensification accelerated aluminum dissolution and pit formation, thereby lowering the apparent *R*_ct_. These effects were most clearly observed at 200 mA·cm^–2^ (Figure 4i–l), where Nyquist plots retained well-defined semicircular shapes. In these cases, *R*_s_ remained constant while *R*_ct_ progressively decreased due to ongoing anodic dissolution in the absence of insulating surface deposits. In some experiments at lower CDs (Figure 4a,e,h), the *R*_ct_ initially decreased, similar to other experiments with progressively intensifying CDs. However, *R*_ct_ subsequently increased during later cycles, which is attributed to the near-complete electrode surface coverage by deposited materials, which hindered charge transfer via diffusion limitations. This trend was particularly strong in experiments without PR, where R_s_ increased substantially when electrode surfaces got covered faster than in experiments with PR.

#### 3.3.2 Ohmic Overpotentials Increased Electrode Potentials as Revealed by Statistical Correlations

Figure 5 shows strong linear positive correlations between measured increases in electrode potential and ohmic overpotentials quantified via EIS across 12 experimental conditions involving three CDs and four PR intervals, including a control without PR. This suggested that the rise in potential under galvanostatic conditions was predominantly governed by resistive ohmic losses within the system and was confirmed by a two-tailed paired sample *t*-test (*p* > 0.05, SI Table S15).

**Figure 5:**
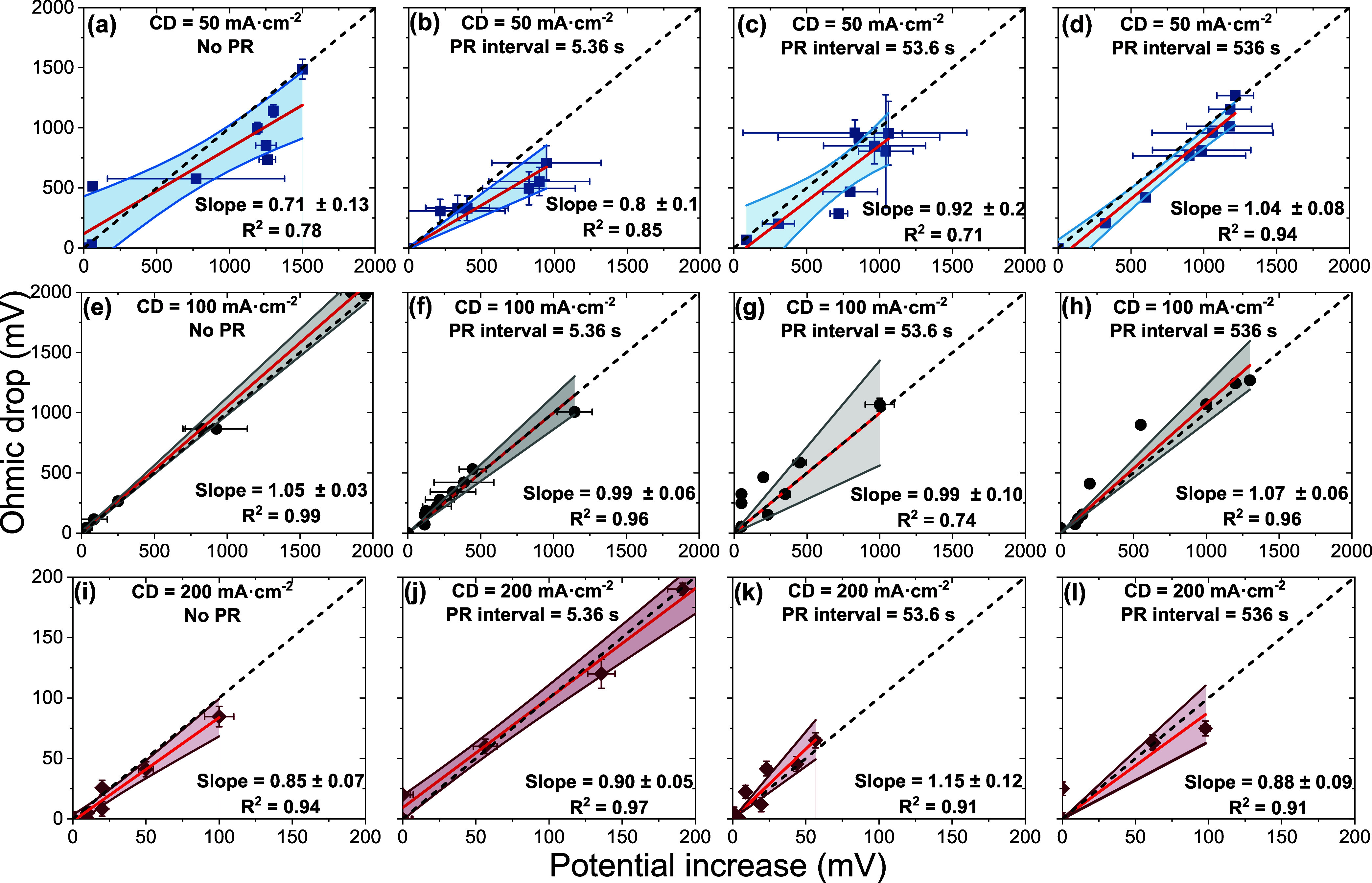
Strong positive linear correlations between the measured increase in potentials during electrocoagulation and corresponding ohmic overpotentials determined using EIS. The operational parameters for CD = 50 mA·cm^–2^ with (a) no PR, (b) PR interval = 5.36 s, (c) PR interval = 53.6 s, (d) PR interval = 536 s, CD = 100 mA·cm^–2^ with (e) no PR, (f) PR interval = 5.36 s, (g) PR interval = 53.6 s, (h) PR interval = 536 s, CD = 200 mA·cm^–2^ with (i) no PR, (j) PR interval = 5.36 s, (k) PR interval = 53.6 s, (l) PR interval = 536 s. Each data point represents the cell potential at the end of a given cycle, plotted against the ohmic drop calculated using the solution resistance (*R*_s_) measured immediately after that cycle. The solid black line represents the linear regression fit, while the black dashed line denotes the line of equality (i.e., a slope of 1), indicating a scenario in which the entire increase in potential could be solely attributed to ohmic losses. The colored area represents a 95% confidence interval.

Across the full data set, slope values ranged from 0.71 ± 0.13 to 1.15 ± 0.12 mV/mV. However, the magnitude of the potential increase was highest without PR (1500 ± 25 mV), consistent with higher cathodic overpotentials (Figure 3a) and trends observed in corresponding Nyquist plots (Figure 4a). After PR, potentials dropped slightly to a range between 945 ± 374 mV and 1215 ± 125 mV. At the medium CD (100 mA·cm^–2^), slopes clustered near unity (0.99–1.07) with high correlation coefficients (0.74–0.99), demonstrating that resistive effects were majorly responsible for the potential rise. The magnitude of the potential increase in these cases was more moderate for PR experiments (1090 ± 10 to 1,200 ± 30 mV), again with the highest values corresponding to the no-PR scenario (1950 ± 70 mV). At the highest CD evaluated, slopes again were close to unity (0.85–1.15 mV/mV) with high correlation coefficients (≥0.91). Notably, the absolute values of both the measured potentials and the calculated ohmic overpotentials were an order of magnitude lower than those calculated at the lower current densities (ranging from 56 ± 3 to 191 ± 10 mV). Note reduced *y*-axis scale from 2000 to 200 mV in Figure 5 for 200 mA·cm^–2^.

Small deviations in slopes and correlation coefficients were likely attributable to localized heterogeneities in foulant distribution, transient interfacial conductivity variations, gas bubble formation, or minor contributions from activation and concentration overpotentials due to changes in the electrode–electrolyte microenvironment. Nevertheless, such small variations did not undermine the overarching mechanistic interpretation that higher potentials were quantitatively driven by resistive effects. Consistent statistical agreement between the measured ohmic overpotential and the total potential increase under all PR and current conditions underscored that ohmic resistance, not kinetic or mass-transfer limitations, was the dominant cause of fouling.

### 3.4 Increasing Ohmic Resistances were Caused by Calcite and Brucite Persisting Strongly on the Electrode Surface That Limited Contact between the Electrode and the Electrolyte

To elucidate the chemical composition and bonding of the surface deposits responsible for increased surface resistance, XPS and XRD analyses were conducted on foulants (electrodeposited solids) scraped from the electrode surface after electrocoagulation (Figure 6). XPS survey spectra (SI Figure S5a) revealed the presence of carbon, aluminum, magnesium, calcium, chlorine, sodium, and oxygen. Calcium and magnesium were associated with scale-forming precipitates, while sodium and chlorine were attributed to residual NaCl due to drying of the deposits. High-resolution XPS scans of the Ca 2p region displayed a doublet with a binding energy separation of 3.5 eV, characteristic of Ca–O bonding, indicative of calcium carbonate^60^ (Figure 6b). This assignment was validated by the C 1s spectrum, which exhibited a peak near 288.8 eV corresponding to C = O functionality^61^ (Figure 6a). The Mg 2s region showed a single, well-resolved peak consistent with Mg–O bonding, indicative of magnesium hydroxide^62^ (Figure 6c). These findings were corroborated by XRD, which identified calcite and brucite as the dominant crystalline phases along with halite, all of which were consistent with expected precipitation products under the alkaline microenvironment formed near the cathode during electrolysis^4,63,64^ (Figure 6e). Additionally, a doublet was observed at 134.4 and 136.2 eV in the Sr 3d high-resolution XPS scan, hinting at small amounts of SrSO_4_ ^65^ (Figure 6d), consistent with strontium removal.^5^ These precipitates exhibit low electrical conductivity,^64,66^ whose accumulation would have increased interfacial resistances, corroborating *in situ* EIS measurements.

**Figure 6:**
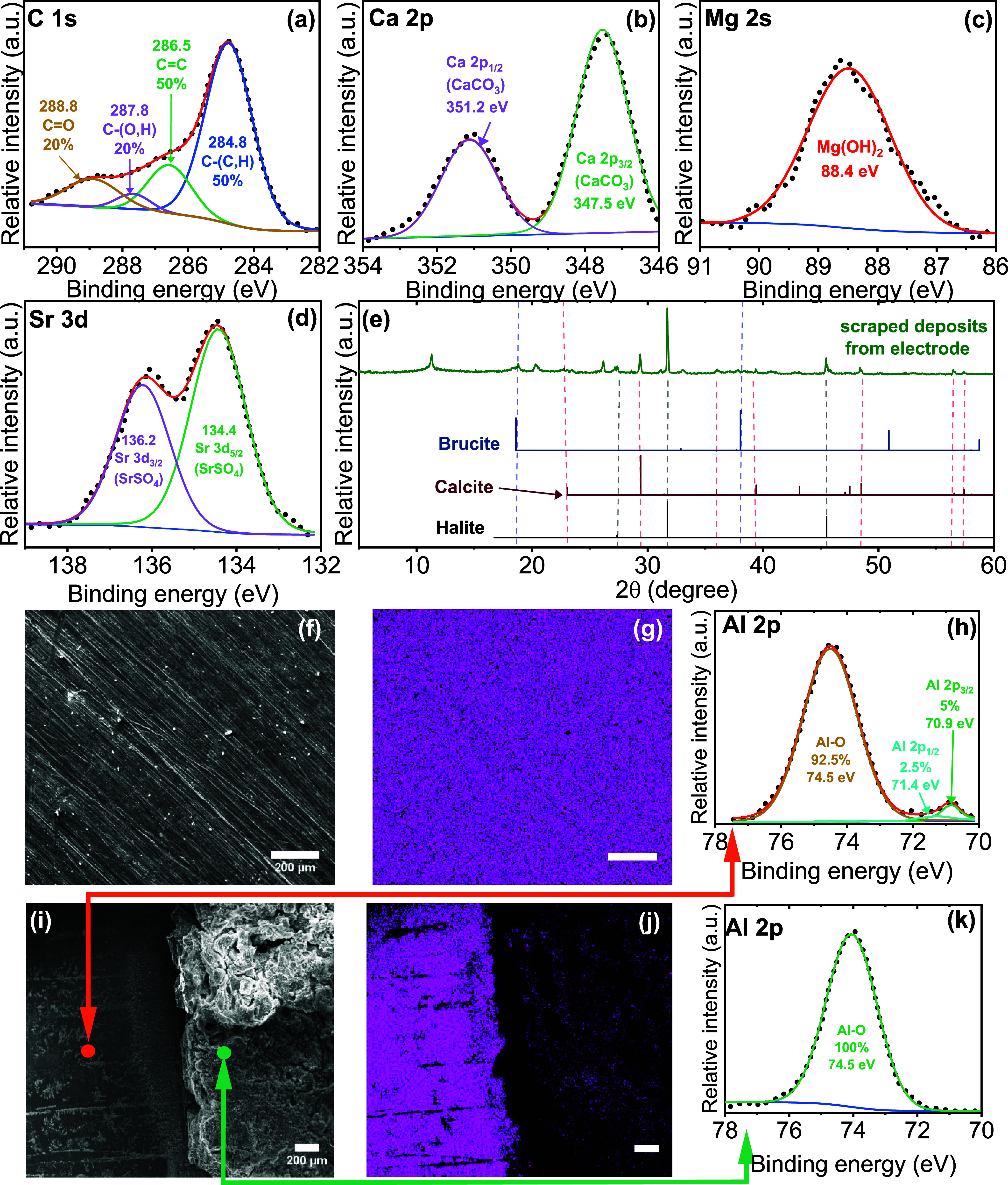
High-resolution XPS scans for (a) C 1s, (b) Ca 2p, (c) Mg 2s, (d) Sr 3d, (e) XRD patterns for electrodeposited solids with supporting stick patterns for brucite, calcite, and halite, electron micrographs of (f) virgin cathode, (i) cathode after electrocoagulation, panels (g) and (j) show corresponding EDS maps, panel (h) shows XPS high-resolution spectra of a point which did not undergo electrolysis due to masking by a nonconductive tape, and (k) high-resolution XPS spectra of a point of aluminum entrapped in thick layer of foulant. Panels (f), (g), (i), and (j) show scale bars of 200 μm.

Surprisingly, aluminum was detected in XPS survey spectra of dislodged foulants despite taking care to only gently remove them and not touching the electrode surface. To investigate this further, EDS and high-resolution XPS were performed on bare electrode surfaces before and after electrocoagulation. EDS spectra of the virgin electrode (Figure 6f–g) confirmed the dominant presence of aluminum, with no detectable calcium or magnesium. Following electrocoagulation, areas protected by masking tape (i.e., pristine electrode) displayed pure aluminum EDS signatures, whereas areas covered with deposited solids showed strong calcium, magnesium, and aluminum signals (Figure 6i–j, SI Figure S6a–c). Given that deposits were thick (93 ± 23 μm), it was improbable that this signal originated from the underlying electrode substrate, because the EDS effective penetration depth is only ∼2 μm at 30 keV excitation.^67^ High-resolution XPS of the Al 2p region for dislodged foulants revealed a single peak associated with Al–O bonding, suggesting the presence of aluminum hydr­(oxide) and ruling out the presence of metallic aluminum entrapped within the fouling layer, otherwise dominated by calcite and brucite. Conversely, a region beneath the nonconductive tape mask showed two peaks at 71.4 eV corresponding to metallic aluminum and 74.5 eV corresponding to aluminum (hydr)­oxides (Figure 6h,6k). These findings supported a mechanism wherein aluminum hydrolysis products nucleate heterogeneously on growing calcium and magnesium deposits. We hypothesized that these precipitates became physically entrapped within the growing foulant layer. Further quantification of this entrapped aluminum within the (electro)­deposits is reported next.

### 3.5 Aluminum Entrapment within the Fouling Layer Reduced Its Concentrations in the Bulk Electrolyte (i.e., Decreased Coagulant Dosing) and Apparent Faradaic Efficiency

Figure 7 depicts bulk aluminum concentrations measured at the conclusion of electrocoagulation experiments (CL = 32,160 C·L^–1^). For all monovalent electrolyte controls, aluminum concentrations surpassed Faraday’s law predictions (3000 mg·L^–1^), reaching 3855 ± 64, 4165 ± 207, and 4375 ± 193 mg·L^–1^ at 50, 100, and 200 mA·cm^–2^, respectively. This apparent “super-Faradaic” behavior was attributed to chemical dissolution of (i) anodic aluminum via chloride-induced pitting corrosion and (ii) cathodic aluminum under the locally elevated pH conditions generated via water reduction (SI Reactions R4 and R5).^5,68^ Moreover, bulk aluminum concentrations were strongly positively correlated with CD. At higher CDs, anodic overpotential and pH at the localized cathodic microenvironment increased, both of which accelerate corresponding chemical corrosion processes.^5,68^ Similar results were obtained for produced water experiments without PR.^5^

**Figure 7:**
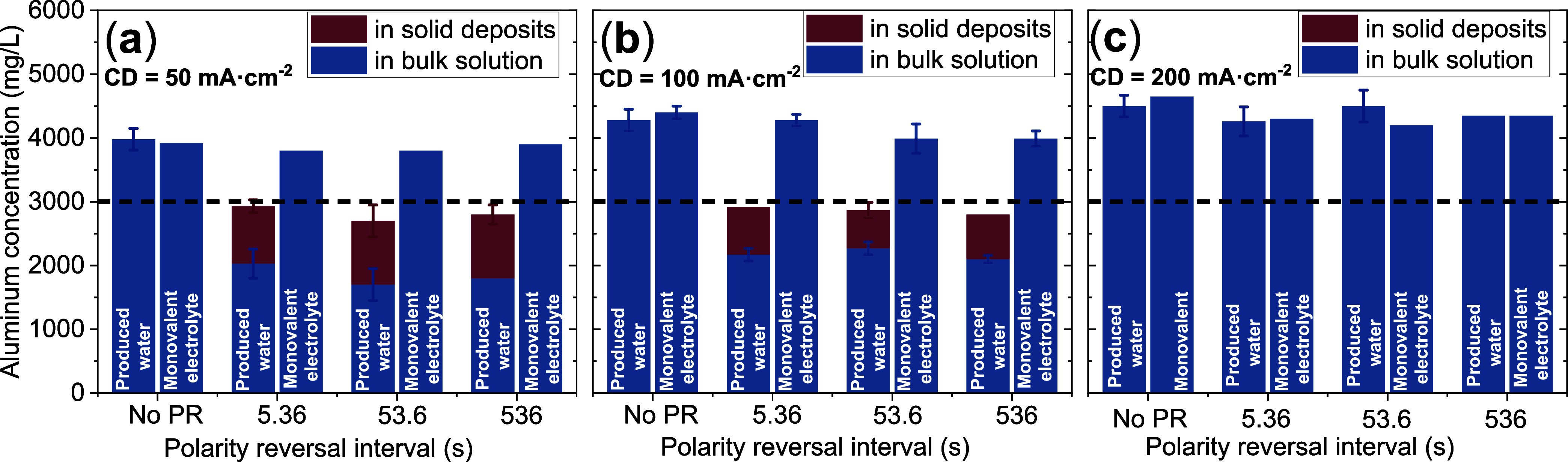
Aluminum concentrations in the bulk suspension (blue) and in the solid (electro)­deposits (brown) at no PR and three PR intervals (5.36 s, 53.6 and 536 s) for (a) CD = 50 mA·cm^–2^, (b) 100 mA·cm^–2^, and (c) 200 mA·cm^–2^ at the end of each electrocoagulation experiment (CL = 32,160 C L^–1^). The horizontal dotted line in each figure represents the value predicted by Faraday’s law (3000 mg L^–1^).

Notably, in contrast to the monovalent electrolyte, aluminum concentrations in experiments with PR in produced water at CD = 50 and 100 mA·cm^–2^ were <3000 mg·L^–1^ irrespective of PR intervals (i.e., apparently sub-Faradaic). However, experiments at CD 200 mA·cm^–2^ with PR generated aluminum concentrations consistently >3000 mg·L^–1^ (i.e., apparently super-Faradaic). This discrepancy was hypothesized to (i) arise from competing side reactions at elevated anodic potentials (e.g., oxidation of adsorbed H_2_ ^23^ and oxidation of chloride and water^4,39^ ), which reduce the efficiency of aluminum dissolution; and (ii) hindered (and slower) migration of aluminum ions, resulting in their hydrolysis, nucleation, and entrapment within fouling layers on electrode surfaces^69^ as in previous publications.

However, as detailed in Sections 3.2.1 and 3.2.2, where anodic potential trends were analyzed, the measured potential increase during PR experiments was attributed predominantly to increasing ohmic overpotential, which utilized additional potential energy, as opposed to activation overpotential. Therefore, even though side reactions were thermodynamically feasible, they were unlikely to occur at the recorded potentials. However, bubbles visually originated from the anode in all experiments with both electrolytes, likely due to anodic hydrogen evolution arising from localized breakdown of the passive film at regions of direct electrolyte–electrode contact. The high potential difference (1.28 V at pH 6.4) between aluminum oxidation and hydrogen evolution makes anodic hydrogen evolution feasible under elevated chloride levels, which is proven to cause pits by local rupture of passive films.^70–72^ Indirect evidence of direct contact of electrode and electrolyte was provided by the presence of crystallographic pits (SI Figure S7).^73^ Moreover, as this is a non-Faradaic chemical reaction, it did not consume current and therefore did not contribute to a reduction in Faradaic efficiency.^72^ Nevertheless, superfluous hydrogen evolution was comparable in the monovalent electrolyte and produced water at 200 mA·cm^–2^, where apparent Faradaic efficiency remained high (>100%), indicating that it did not electrochemically compromise aluminum dissolution efficiency in these experiments.^70,71^ However, it may have contributed to enhanced aluminum dissolution by non-Faradaic pathways, contrary to the possible side reactions reducing the apparent Faradaic efficiency as reported in several PR studies,^1,22,23^ which is another major novel contribution of this manuscript.

To make robust conclusions about restricted migration of electrodissolved aluminum ions, we quantified the aluminum content entrapped within solid (electro)­deposits after gently scraping them from both electrodes and digesting the solids at high temperature and high pressure in closed vessels using nitric acid in a microwave digester (SI Section S4) via inductively coupled plasma–mass spectrometry^74^ and are shown as brown segments in Figure 7. Accounting for aluminum’s presence in both the bulk suspension (as flocs) and entrapped within the fouling layer, its overall concentrations approached the Faradaic value across all PR intervals for 50 and 100 mA·cm^–2^. The absence of super-Faradaic dissolution in these PR experiments, as compared to a monovalent electrolyte, was attributed to (i) a reduced active surface area for anodic dissolution due to insulating deposit layers and (ii) a reduced cathodic area for chemical dissolution due to fouling. At the highest CD investigated (200 mA·cm^–2^), Faradaic efficiencies in produced water were comparable to the monovalent electrolyte, regardless of PR intervals. This is attributed to minimal fouling due to enhanced hydrogen evolution-induced surface scouring that is supported by electrode photographs post-electrocoagulation (SI Figure S2 k-l).

### 3.6 Modest Removal of Divalent Ions and Better Removal of Silicon and Boron

Silicon was well-removed across all experiments (97 ± 2%) (Figures 8a,8b), similar to our recent study, and was due to the formation of aluminosilicate flocs.^5^ FTIR showed a peak in the region 1018–975 cm^–1^ consistent with Al–O–Si stretching vibrations (SI Figure S9).^75^ Moreover, a wide band near 1080 cm^–1^ indicated an asymmetric Si–O–Si stretching band. Wavenumbers of 590 and 1630 cm^–1^ supported Al–OH stretching and deformation of adsorbed molecular water on Si–O–Si, respectively.^75^ Boron removal was intermediate (54 ± 2%) and attributed to its complexation with aluminum hydroxide floc surfaces.^34,76,77^ Calcium and strontium were poorly removed in terms of percentages (6.8 ± 1.5% and 2.9 ± 0.2%, respectively), whereas magnesium was moderately removed (24 ± 2%). However, the absolute quantity of calcium and magnesium removed was substantial, 252 ± 57 and 194 ± 26 mg·L^–1^, respectively, far exceeding the removal reported in low salinity simulated groundwater.^4^ Low strontium removal was attributed to the produced water’s low inorganic carbon content.^78^ Nonetheless, high-resolution XPS analysis of the electrode surface revealed the presence of strontium sulfate (Figure 6d), indicating lower concentrations that were not reflected in the bulk aqueous phase measurements. None of these species exhibited any discernible removal trends with CD or PR interval. Monovalent anions (e.g., Cl^–^) and cations (e.g., Na^+^, K^+^) were not appreciably removed, similar to conventional coagulation.

**Figure 8:**
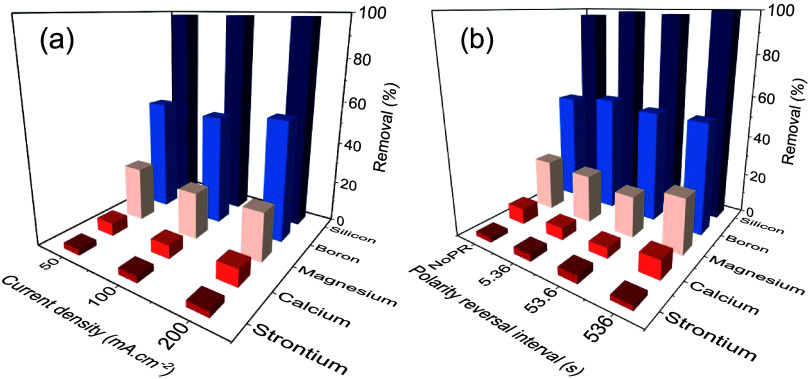
Removal of calcium, magnesium, strontium, boron, and silicon with changing (a) current density and (b) polarity reversal interval. Samples were taken at the end of each experiment, i.e., CL = 32,160 C·L^–1^ and aluminum dose ∼3000 mg·L^–1^.

## 4 Implications

The four major novel findings of this research are that during galvanostatic electrocoagulation with PR, (electro)­deposited solids: (i) caused fouling that was almost solely due to ohmic overpotentials ruling out any role of even thermodynamically feasible electrochemical side reactions, (ii) disrupted electrode–electrolyte contact rendering direct electrochemical means of fouling mitigation ineffective, (iii) increased electrode potentials that were quantitatively linked to interfacial resistance (ohmic overpotentials) by intermittent EIS measurements, and (iv) were dislodged effectively only above a threshold CD attributed to higher hydrogen bubble generation rate. Hence, foulant removal must rely on purely mechanical means, such as scraping/brushing electrode surfaces after placing the system offline or indirect electrochemical *in situ* scouring by hydrogen gas bubbles. Intermittent EIS measurements represent a novel *in situ* diagnostic tool and provide proof-of-concept for real-time quantitative monitoring of electrode fouling, which could possibly be extended to conductive membranes.^47,79^ This also revealed quantitatively that the additional potential energy did not enable even thermodynamically feasible side reactions by overcoming the activation barrier (activation overpotential) but was instead fully utilized for compensating the restricted ionic mobility/reduced surface area (ohmic overpotential) to maintain galvanostatic operation. Reasons underlying the limited effectiveness of PR in operationally controlling fouling over the entire range of experimental conditions investigated were dependent on CD. At low CDs, PR only seemed to redistribute foulants across the electrode surface, whereas at high current density, foulant accumulation was low even for constant polarity, negating the need for PR. Hydrogen bubble evolution was identified as the dominant pathway in mitigating cathodic reaction-driven electrode fouling with only negligible assistance from anodic corrosion underlying foulant deposits. These findings enable an informed evaluation of PR, avoiding its application as a black-box operational strategy and for optimizing electrocoagulation systems, particularly in very hard feedwaters (i.e., high concentrations of divalent cations), where fouling dynamics are still relatively unexplored. In our experiments, magnesium, calcium, and strontium were removed to moderate-low extents through electrodeposition (30–40, 5–10, and 5–10%, respectively), boron and silicon were well-removed via floc-uptake (∼60 and >95%, respectively), and turbidity was very well removed by enmeshment and sweep flocculation (>95%) (SI Section S9). These water quality results further position electrocoagulation as a promising treatment technology but were not pursued in detail in this manuscript because associated mechanisms have already been established.^3–5,76^ It is emphasized that batch experiments were conducted herein to predominantly focus on electrochemical mechanisms rather than hydrodynamic effects on fouling. Therefore, longer interval experiments in continuous-flow reactors are needed, incorporating the synergistic role of shear to better understand electrode longevity and fouling under more realistic operating conditions.^4,24,68,81^ Nevertheless, batch results point to the need for optimizing CD under site-specific conditions to electrochemically scour surface deposits and control fouling by generating hydrogen bubbles at a sufficient rate (separate from hydrodynamics), especially when influent water quality fluctuates dynamically.^15,21^

## Supplementary Material



## Notes

Feed (raw produced water) and treated (post-electrocoagulation) water quality, electrode composition, experimental setup and analytical procedures; electrochemical and surface characterization of electrodes and surface characterization of flocs using XPS, EDS, XRD, FTIR, and SEM; interfacial (electro)­chemical reactions; EIS details including fitting parameters for data at each CD and PR interval; glossary of relevant electrochemical terms and reactions; short review of the PR literature, pictures of pristine and fouled electrodes; and Nyquist plots for all experiments (PDF)
