# Supplementary material for: Ohmic Losses Dominated Electrode Fouling during Long-Term Aluminum Electrocoagulation of Hypersaline and Divalent Cation-Rich Oilfield-Produced Water
Source: ACS ES T Eng. 2025 Oct 17;5(12):3519–32. doi: 10.1021/acsestengg.5c00628 (PMC12706792; doi:10.1021/acsestengg.5c00628)
Supplement: Supplementary file 1 [file ee5c00628_si_001.pdf]

Supporting Information for

Ohmic Losses Dominated Electrode Fouling During Long-Term Aluminum Electrocoagulation of Hypersaline and Divalent Cation-Rich Oilfield Produced Water

Sanket Joag[a], Jonathan Kiesewetter[b], Shankararaman Chellam[a,c,*]

[a] Department of Civil & Environmental Engineering and Department of Chemical Engineering, Texas A&M University, College Station, TX 77843-3136, United States

[b] Process, Water & Heat Transfer Engineer, Global Production – Facilities Engineering, ConocoPhillips, 925 N. Eldridge Parkway, Houston, TX 77079, United States

[c] Department of Chemical Engineering, Texas A&M University, College Station, TX 77843-3122, United States

* Corresponding author: Shankararaman Chellam. Zachry Department of Civil & Environmental Engineering, Texas A&M University, College Station, TX 77843. Phone: (979) 458 5914; chellam@tamu.edu

Table of contents

Section	Title	Page number
S1	Produced water composition	3
S2	Aluminum electrode composition	4
S3	Electrochemical reactor setup	5
S4	Materials and methods	6
S5	Theory and calculations for overpotentials	12
S6	Activities and electrochemical reactions	15
S7	Literature review	17
S8	Electrode optical images	18
S9	EIS fitting parameters and hypothesis testing for ohmic and total overpotentials	20
S10	Surface characterization for (electro)deposits and flocs	34
S11	Operational results for contaminant removal	35

Section S1. Produced water composition

Table S1. Composition of produced water and synthetic NaCl solution.

**Parameters**	**Units**	**Produced water**
pH	-	6.4±0.1
Turbidity	NTU	85±3
Total Dissolved Solids (TDS)	mg/kg	120,000±3,200
Conductivity	mS/cm	130
Total Organic Carbon (TOC)	mg/L	59.8±2.9
Dissolved Organic Carbon (DOC)	mg/L	52.4±1.2
Silicon	mg SiO_2/L	35±2
Lithium	mg/L	35±1
Boron	mg B/L	233±22
Sodium	mg/L	42,000
Magnesium	mg/L	790±22
Manganese	mg/L	2.1±0.1
Aluminum	µg/L	30±5
Barium	mg/L	6±1
Potassium	mg/L	1,154±78
Calcium	mg/L	3,530±127
Iron	mg/L	22±2
Strontium	mg/L	1,048±13
Sulfate	mg/L	180±5
Alkalinity	mg $CaCO_3$/L	65±5
Chloride	mg/L	78,000±2,000

Section S2. Aluminum electrode composition

Table S2. Composition of aluminum electrodes used in the study provided by the manufacturer.

Content	%
Al	99.28
Fe	0.46
Si	0.11
Cu	0.07
Mn	0.006
Mg	0.006
Cr	0.001
Ni	0.004
Zn	0.005
Ti	0.02

Section S3. Electrochemical reactor setup

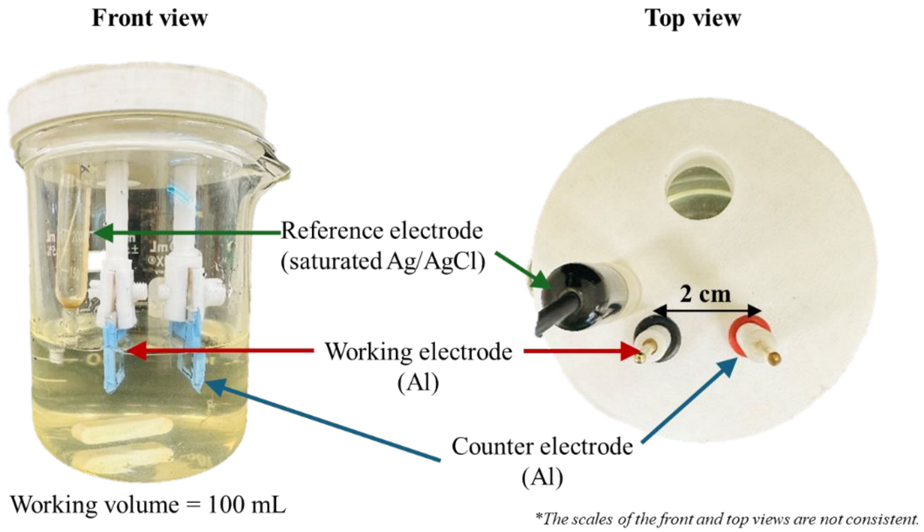

Figure S1. Electrode setup for electrocoagulation (with and without polarity reversal). The batch reactor was 250 mL beaker with 100 mL working volume.

Section S4. Materials and methods

1. Produced water characterization

Numerous water quality analyses were employed to measure the raw and treated water quality. pH and conductivity were measured using probes (Orion 9146BN and HACH CDC401, respectively). The turbidity was measured using HACH 2100 N turbidity meter. Dissolved organic carbon and total organic carbon was measured using Shimadzu TOC-L analyzer by separately calculating total carbon and inorganic carbon and subtracting them to get inorganic carbon.

Ionic and chemical compositions were thoroughly investigated using Inductively Coupled Plasma-Mass Spectrometry (ICP-MS) and colorimetry using HACH DR6000 spectrophotometer. The samples were acidified by adding 70% HNO_3 in a 1:1 ratio before digesting/dissolving the suspended particles in the samples, by heating the solution in closed Teflon vessels at 180°C for 15 minutes in a microwave oven (CEM MARS 6, Matthews, North Carolina). The digested samples were diluted to 2% HNO_3 acid matrix before analyzing with quadrupole ICP-MS (q-ICP-MS; ELAN DRC II, PerkinElmer SCIEX) for detection of cations. ICP-MS equipped with a collision cell pressurized with NH_3 (q-ICP-MS; ELAN DRC II, PerkinElmer SCIEX) for detection of cations. Improved analysis of Fe was facilitated by exploiting ion-molecule reaction with NH_3 with a gas flow rate of 1 mL min^{-1}.

Aluminum concentrations were determined colorimetrically using the Eriochrome cyanine R (ECR) method (HACH method #8326). Each sample was digested with 70% HNO_3 by heating the solution in closed Teflon vessels at 200ºC for 15 minutes after ramp time of 15 minutes. Prior to analysis, samples were brought back to near-neutral pH and diluted appropriately with ultrapure water to ensure the aluminum concentration was within the method range. The color intensity of the Al–ECR complex is proportional to the concentration of aluminum present in the sample. The absorbance of the complex was measured at a wavelength of 535 nm using a UV–Visible spectrophotometer (HACH DR 6000) with 1 cm path-length cells. All measurements were performed at least in duplicates, and the average values were reported with corresponding standard deviations.

Sulfate was measured using USEPA SulfaVer 4 method (HACH method #8051). Sulfate in the sample reacts with barium present in the kit to form barium sulfate as a precipitate. The amount of turbidity is proportional to the amount of sulfate and is measured by colorimetry at 450 nm. Finally, TSS and TDS were measured according to the standard protocols (Methods #2540C and #2540D for TDS and TSS respectively)[1].

2. Electrical potentials and data analysis during polarity reversal experiments

Polarity reversal (PR) was implemented using the *Chronopotentiometry* on a Gamry electrochemical workstation (1010E, Gamry Instruments). The working electrode (aluminum) alternated polarity at defined intervals to operate sequentially as anode and cathode. Potentials were recorded versus an Ag/AgCl reference electrode during each phase, yielding anodic and cathodic potential profiles, respectively. Three current densities were tested: 50, 100 and 200 $mA \cdot cm^{-2}$. These current densities are higher than previously employed for aluminum electrocoagulation to achieve higher charge loading in shorter timeframes, specifically for targeting very high charge loadings. Three PR switching intervals were investigated: 5.36 seconds (corresponding to a full cycle of 10.72 seconds), 53.6 seconds (full cycle of 107.2 seconds), and 536 seconds (full cycle of 1072 seconds). At a current density of 50 $mA \cdot cm^{-2}$, a reversal period of 1072 s corresponds to an aluminum dose of 100 $mg \cdot L^{-1}$ as Al^{3+}. Based on this relationship, polarity reversal intervals were chosen to represent equivalent aluminum doses of 0.5, 5, and 50 $mg \cdot L^{-1}$ at 50 $mA \cdot cm^{-2}$, and these same intervals were maintained at higher current densities for consistency. For experiments conducted without polarity reversal, galvanostatic program at three fixed current densities (CDs) of -50, -100, and -200 $mA \cdot cm^{-2}$ was implemented. In case of experiments without PR, a negative CD was applied to record cathodic potentials and corresponding ohmic overpotential using electrochemical impedance spectroscopy. The total charge loading in all the experiments (with and without PR) was maintained at 32,160 C/L across all experiments, resulting in different total durations based on the applied CD. Accordingly, the number of potential and current data points recorded at 1 data point per second was 32,160, 16,080, or 8,040, depending on the CD.

Due to frequent polarity switching (30, 300, or 3,000 cycles depending on PR interval), the raw data exhibited substantial fluctuations. To extract representative electrochemical trends, the potential data were processed in two stages. First, within each full cycle, the half-cycle in which the initial anode remained anodic (i.e., 5.36 s, 53.6 s, or 536 s) was isolated, and the anodic and cathodic potentials were averaged for that interval. Then, to smooth transient variations and facilitate comparative analysis, the averages were further computed across 100 cycles (for the 5.36 s PR), 10 cycles (for 53.6 s PR), or a single cycle (for 536 s PR). These processed values were then used to construct the anodic and cathodic potential profiles presented in the results. A custom Python script was developed and executed in the Google Colab environment to automate data extraction and averaging.

```
Import pandas as pd
from google.colab import files

uploaded = files.upload()
file_name = list(uploaded.keys())[0]
df = pd.read_excel(file_name)
df_numeric = df.apply(pd.to_numeric, errors='coerce')

serial_numbers = []
odd_block_averages = []
even_block_averages = []

block_number = 1

for I in range(0, len(df_numeric), block_size):
    # Extract a block of 10 rows
    block = df_numeric.iloc[i:I + block_size]
    odd_block = block[(block['Pt'] >= 1) & (block['Pt'] <= 5)]
    if not odd_block.empty:
        odd_avg = odd_block['Vf'].mean()
        odd_block_averages.append(odd_avg)
    else:
        odd_block_averages.append(None)

    even_block = block[(block['Pt'] >= 6) & (block['Pt'] <= 10)]
    if not even_block.empty:
        even_avg = even_block['Vf'].mean()
        even_block_averages.append(even_avg)
    else:
```

```
        even_block_averages.append(None)
    serial_numbers.append(block_number)
    block_number += 1

results_df = pd.DataFrame({
    'Repetition Number': serial_numbers,
    'Odd Block Average (1-5)': odd_block_averages,
    'Even Block Average (6-10)': even_block_averages
})

output_file = 'anodic_cathodic_block_averages_fixed.xlsx'
results_df.to_excel(output_file, index=False)

files.download(output_file)
```

```
import pandas as pd
import os

from google.colab import files
uploaded = files.upload()

for file_name in uploaded.keys():
    data = pd.read_excel(file_name)
    num_entries = len(data)

    group_size = 10 if num_entries == 300 else 100 if num_entries == 3000
else None

    if group_size is None:
        print(f"Unsupported number of entries ({num_entries}) in file:
{file_name}. Skipping.")
        continue


    data = data.apply(pd.to_numeric, errors='coerce'
    averaged_data = data.groupby(data.index // group_size).mean()

    base_name, ext = os.path.splitext(file_name)
    new_file_name = f"{base_name}_FINAL{ext}"

    averaged_data.to_excel(new_file_name, index=False)
    print(f"Processed file saved as: {new_file_name}")
```

3. Potentiodynamic polarization

Potentiodynamic polarization was performed on both the electrodes before and after the EC experiment. The open circuit potential (OCP) was measured for 300 s before each experiment. The potential started from -0.5V vs. OCP and measured until +0.5V vs. OCP. The scan rate of 10 mV/s was maintained in all the experiments.

4. Electrochemical impedance spectroscopy:

EIS experiments were conducted in potentiostatic mode, involving the application of a small AC perturbation potential while monitoring the current response. A sinusoidal root mean squared potential of 10 mV rms was applied at OCP over a frequency range of 100 kHz–100 mHz. The linearity, causality, and stability of the system were verified using the Kramers-Kronig test after each experiment and only the experiments which followed the criteria were considered[2]. The circuit models were constructed using Gamry Echem Analyst (Gamry Instruments) and fitted with the same software. The uncompensated ohmic resistance was obtained after fitting a Nyquist plot to an equivalent electrical circuit and extracting the resistance from the high frequency range[3].

5. XPS

The surface functional groups and speciation for electrodeposits, flocs and electrodes were analyzed using a PHI 5700 X-ray photoelectron spectrometer with a monochromatic Al Kα X-ray source (hv = 1486.7 eV). The collected flocs were washed with ultrapure water and centrifuged several times to reduce chloride interference. Survey scans for elemental composition over the entire range (0-1400 eV), and high-resolution scans for C 1s, O 1s, Al 2p, Si 2p, Ca 2p, Mg 2s, and Sr 3d were taken based on the need. Charge effects were accounted for by calibrating C 1s binding energy to 284.8 eV, as detailed previously[4,5]. Peak assignments were conducted with reference to the NIST XPS database.

6. SEM-EDS

Flocs and electrodes were sputter-coated with 10 nm of gold before acquiring images to avoid charging effects. The microscope is equipped with an Oxford energy dispersive x-ray spectroscopy system to identify the elemental composition.

7. XRD

The cathode was dried after the experiment to remove all the moisture using a vacuum desiccator for 24 hours. The electrodeposited solids on the cathode were then gently removed. The solids were then analyzed with an X-ray diffractometer after placing in a sample holder of a two-circle goniometer, enclosed in a radiation-safety enclosure. The X-ray source was a 1kW Cu X-ray tube (λ = 1.54060Å), maintained at an operating current of 40 kV and 25 mA. The diffraction patterns were collected from 5^0 to 80^0 with an angular step size of 0.015^0 2Θ. The International Centre for Diffraction Data (ICDD) database was used to identify the peaks.

8. ATR-FTIR

Flocs were mounted on ITX coated diamond monolithic assembly to acquire the vibrational spectra in ATR (attenuated total reflection) mode for the wavenumber range of 4000-600 cm^{-1}. A background spectrum was collected before each sample, and measurements were taken as a triplicate of 128 scans, each with a resolution of 4 cm^{-1}. The recorded spectra were averaged and corrected for the baseline and the wavelength dependence of ATR.

Section S5. Theory and calculations for overpotentials

Overpotential and types of overpotentials[6,7]:

In electrochemical systems, overpotential refers to the excess potential that must be applied beyond the thermodynamic equilibrium potential to sustain a desired rate of electrochemical reaction. This deviation arises due to various kinetic and transport limitations at the electrode–electrolyte interface and within the bulk electrolyte.

1. Activation overpotential - Originates from the intrinsic energy barrier associated with charge transfer reactions and reflects the kinetics of electron exchange at the electrode surface; it is quantitatively described by the Butler–Volmer equation.
2. Concentration overpotential - Originates when there is a deviation in the concentration of electroactive species at the electrode surface relative to the bulk, typically under conditions of high current density where mass transport cannot replenish reactants or remove products rapidly enough.
3. Diffusion overpotential - Accounts for the potential gradient caused by finite diffusion rates of species through the diffusion layer.
4. Ohmic overpotential - results from resistance to ionic conduction in the electrolyte and electronic conduction through electrodes or other cell components.

Potentiodynamic polarization[6]:

Polarization refers to the deviation of an electrode from its steady-state potential, either in the positive or negative direction. Potentiodynamic polarization involves varying the electrode potential at a fixed scan rate while measuring the corresponding current response. The resulting polarization curve is commonly presented as a semi-logarithmic plot of current density versus potential (log I–V).

Under typical conditions, log I increases with potential due to activation polarization. However, for certain metals such as aluminum in aerated solutions, log I may remain constant or even decrease despite increasing potential. This behavior arises from the formation of a protective oxide film on the metal surface, which increases the barrier to dissolution. The phenomenon is manifested as a plateau region in the log I–

V curve. In highly aggressive environments, such as concentrated chloride solutions, chloride ions can penetrate these passive films through vacancy transport mechanisms, thereby disrupting the protective layer and initiating localized corrosion[8].

Calculation of ohmic overpotentials using EIS

The ohmic overpotential were calculated according to ohm's law:

$$ohmic\ overpotential\ (mV) = j\left(\frac{mA}{cm^2}\right) \times R\ (ohm \cdot cm^2)$$

To evaluate the ohmic contribution to the total cell voltage during electrocoagulation experiments, the solution resistance (R_Ω) between the anode and the reference electrode was estimated based on the bulk conductivity of the electrolyte and the geometric configuration of the electrochemical cell. Under uniform current distribution and well-defined electrode geometry, R_Ω can be calculated using Ohm's law adapted for ionic conduction:

$$R_\Omega = l / (\sigma \times A)$$

where:

- R_Ω is the solution resistance in ohms (Ω),
- l is the distance between the working (anode) and reference electrodes (cm),
- σ is the bulk electrical conductivity of the solution (S/cm), and
- A is the cross-sectional area available for current flow (cm^2).

In this study, the conductivity of the electrolyte (produced water) was measured to be approximately 130 mS/cm. The distance between the anode and the reference electrode was approximately 0.7 cm, and the estimated cross-sectional area for current flow was 2 cm^2. Substituting these values into the equation, the solution resistance is calculated as ~2.7 $\Omega\cdot cm^2$. This value reflects the uncompensated resistance encountered by the current flowing between the anode and the reference electrode and was used as a

baseline to interpret electrochemical impedance spectroscopy (EIS) results and to distinguish ohmic losses from interfacial or kinetic contributions

Section S6: Activities and electrochemical reactions

The potentials at which electrochemical reactions occur in different electrolytes will differ depending upon the activities of the ions in the solution. This is governed by the Nernst equation.

$$U = U^{\circ} - \frac{RT}{nF} ln \prod a_i^{s_i} \quad (1)$$

U: Electrode potential under non-standard conditions (volts, V)

U°: Standard electrode potential (V) at standard conditions

R: Universal gas constant (8.3145 J mol^{-1} K^{-1})

T: Absolute temperature (K)

n: Number of electrons transferred in the half-reaction

F: Faraday constant (96,485 C mol^{-1})

a_i: Activity coefficient

Possible electrochemical reactions at cathode

Reaction	Half-Reaction	Standard E° (V vs Ag/AgCl)	Nernst Equation	
Aluminum dissolution	$Al \rightarrow Al^{3+} + 3e^-$	–1.857	$E = -1.857 + (0.059/3) \times \log_{10}[Al^{3+}]$	(R1)
Water oxidation (OER)	$2H_2O \rightarrow O_2 + 4H^+ + 4e^-$	+1.032	$E = 1.032 - 0.059 \times pH$	(R2)
Chloride oxidation	$2Cl^- \rightarrow Cl_2(g) + 2e^-$	+1.161	$E = 1.161 + 0.059 \times \log_{10}([Cl^-]^2 / p_{(}Cl_{2)})$	(R3)

Possible electrochemical reactions at cathode

**Reaction**	**Half-Reaction**	**Standard E^0 (V vs Ag/AgCl)**	**Nernst Equation**	
Water reduction	$2H_2O + 2e^- \rightarrow H_2 + 2OH^-$	–0.197	$E = -0.197 - 0.059 \times pH$	(R4)

Reaction	Half-Reaction	Standard E^0 (V vs Ag/AgCl)	Nernst Equation	
Oxygen reduction	$O_2 + 2H_2O + 4e^- \rightarrow 4OH^-$	+0.204	E = 0.204 + 0.059×pH	(R5)

Chemical reactions occurring on anode and cathode:

$$4Al + 3O_2 \rightarrow 2Al_2O_{3(s)} \quad \text{(R6)}$$

$$2Al + 6H_2O \rightarrow 2Al(OH)_{3(s)} + 3H_2 \quad \text{(R7)}$$

$$6H^+ + 2Al \rightarrow 3H_2 + 2Al^{3+} \quad \text{(R8)}$$

$$6H_2O + 2OH^- + 2Al \rightarrow 2[Al(OH)_4]^- + 3H_2 \quad \text{(R9)}$$

Reactions responsible for electrodeposition of Ca and Mg bearing solids:

$$HCO_3^- + OH^- \rightarrow CO_3^{2-} + H_2O \quad \text{(R10)}$$

$$Ca^{2+} + CO_3^{2-} \rightarrow CaCO_{3(s)} \quad \text{(R11)}$$

$$Mg^{2+} + 2OH^- \rightarrow Mg(OH)_{2(s)} \quad \text{(R12)}$$

Theoretical H_2 gas produced (considering H_2 evolution as the only cathodic reaction):

Current density ($mA \cdot cm^{-2}$)	H_2 volume (mL)	H_2 generation rate ($mL \cdot s^{-1}$)	H_2 generation rate/unit area ($mL \cdot s^{-1} \cdot cm^{-2}$)
50	373.5	0.0116	0.0058
100	373.5	0.0232	0.0116
200	373.5	0.0465	0.0232

Section S7: Literature review

Table S3. Literature review showing contrasting results of PR in mitigating electrode fouling

Sr. No	Title	Anode and cathode	Operating conditions	Performance efficiency	References
1	Mitigating Electrode Scaling in Electrocoagulation by Means of Polarity Reversal: The Effects of Electrode Type, Current Density, and Polarity Reversal Frequency	Fe-Fe<br>Al-Al	Synthetic groundwater	Polarity reversal effectively **mitigated** fouling at optimized conditions for Al-Al electrocoagulation but was not effective in mitigating fouling for Fe-Fe system	[9]
2	Performance restoration of iron electrodes by polarity reversal after long-term surface fouling in wastewater electrocoagulation	Fe-Fe	Synthetic secondary effluent<br>pH range 6.5-8.3	Polarity reversal effectively **mitigated** anodic electrode fouling (mostly iron oxyhydroxides)	[10]
3	How does periodic polarity reversal affect the faradaic efficiency and electrode fouling during iron electrocoagulation?	Fe-Fe<br>Al-Al	Synthetic groundwater<br>Synthetic produced water (low salinity)	Polarity reversal **was not effective in mitigating** the fouling of iron electrodes	[11]
4	Effect of polarity reversal on floc formation and rheological properties of a sludge formed by the electrocoagulation process	Fe-Fe<br>Al-Al	Produced water (low salinity)	Polarity reversal effectively **mitigated** fouling	[12]
5	Sustaining efficient production of aqueous iron during repeated operation of Fe(0)-electrocoagulation	Fe-Fe	Synthetic groundwater	Polarity reversal **was not effective in mitigating** the fouling of iron electrodes	[13]

Section S8: Electrode optical images

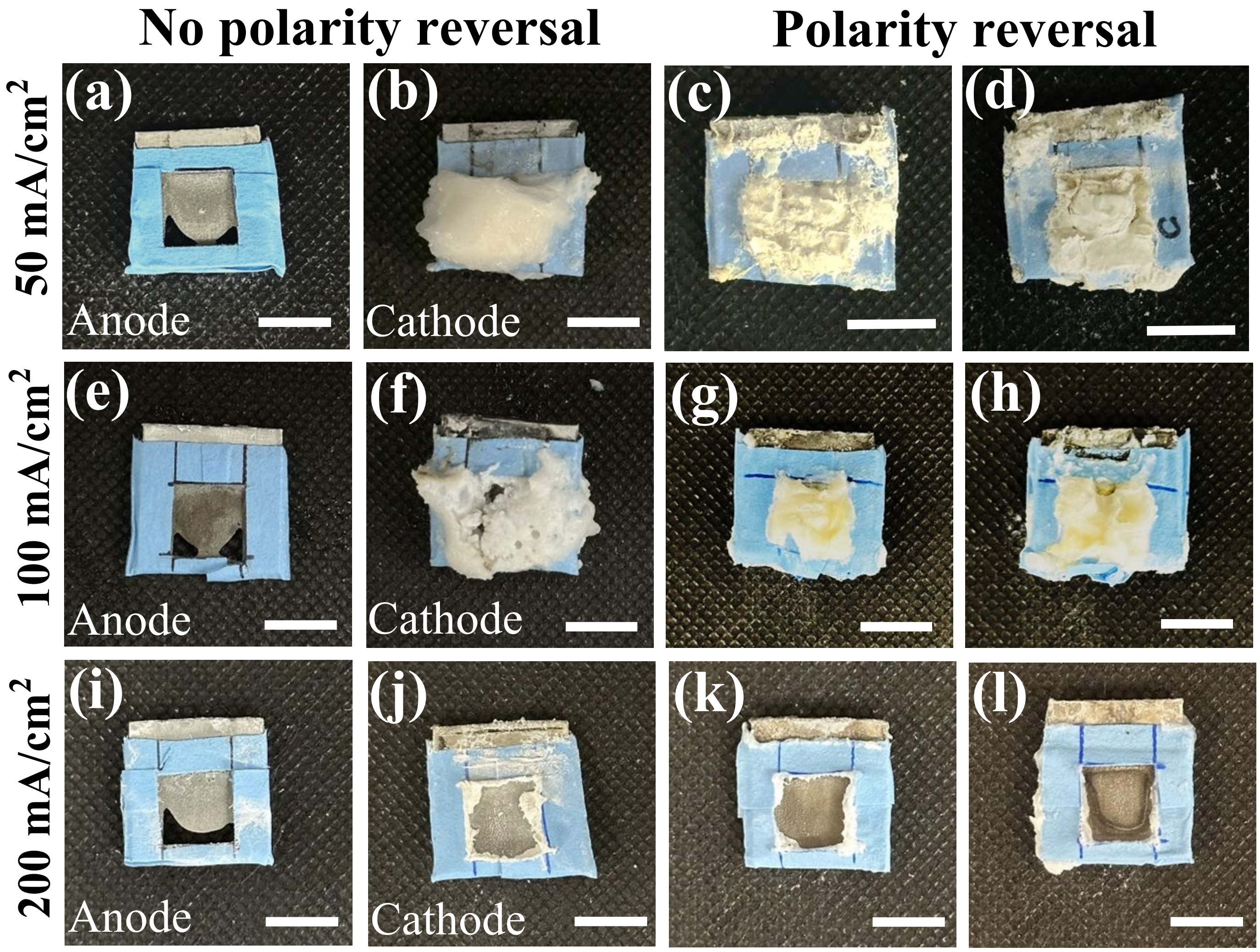

Figure S2. Representative images of the electrodes after CL = 32,160 C·L^{-1}. For experiments with no PR, (a), (e) and (i) represent anodes at the CD = 50, 100 and 200 mA·cm^{-2} respectively. (b), (f) and (j) represent cathodes at the CD = 50, 100 and 200 mA·cm^{-2} respectively. (c) and (d) represent electrodes after PR at 50 mA·cm^{-2}, (g) and (h) at 100 mA·cm^{-2}, (k) and (l) at 200 mA·cm^{-2}. The scale bars represent 1 cm for each image.

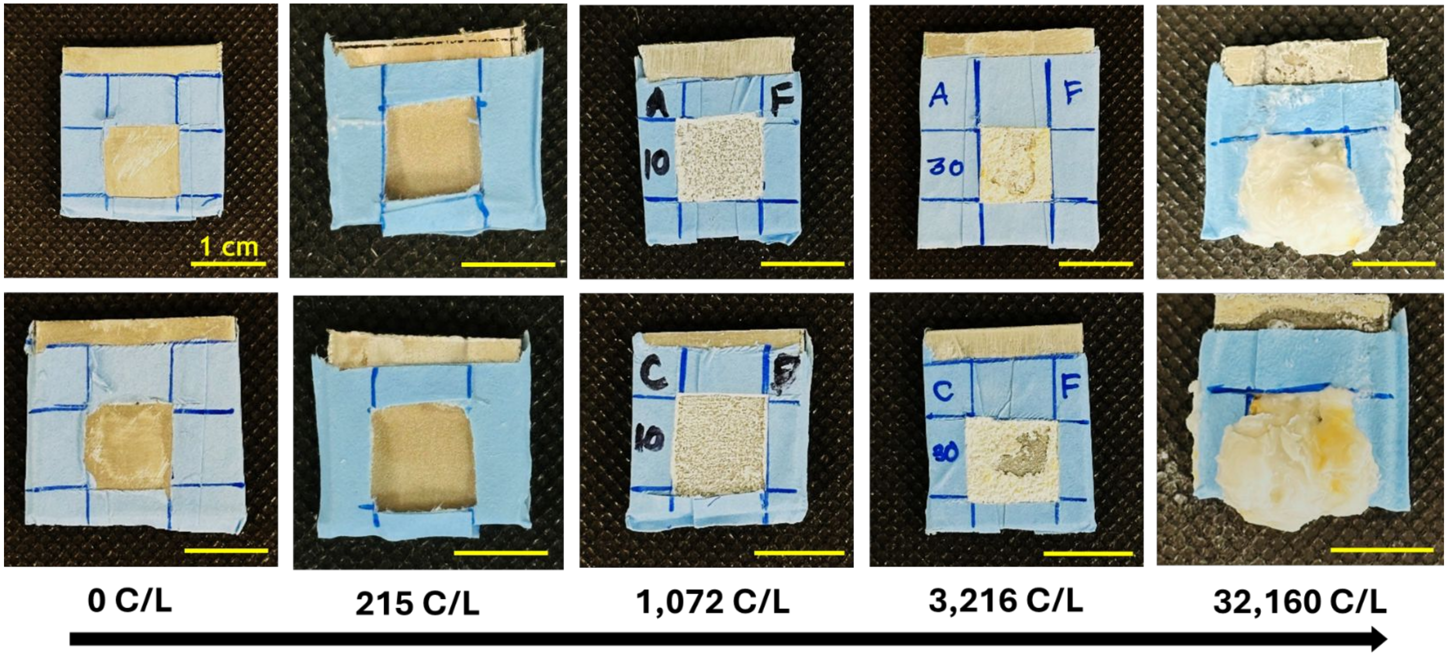

Figure S3. Representative images of electrodes showing a progressive increase in fouling and subsequent reduction in electroactive area. The experiment was performed at CD = 50 $mA \cdot cm^{-2}$ at PR interval = 53.6 seconds.

Section S9: EIS fitting parameters and hypothesis testing for ohmic and total overpotentials

Table S3. Fitting parameters for each charge loading for the operating conditions: CD = 50 mA·cm⁻², no PR

**Charge loading ($C·L^{-1}$)**	**Rs (Ω·cm²)**	**Q1 (S·s^n/cm²)**	**n1**	**R1 (Ω·cm²)**	**Q2 (S·s^n/cm²)**	**n2**	**R2 (Ω·cm²)**
215	2.834 ± 0.03	0.000283 ± 0.00001	0.773 ± 0.006	46.19 ± 0.551	0.188 ± 0.0234	1 ± 0.212	688.7 ± 215
4,288	2.82 ± 0.035	0.00047 ± 0.00003	0.694± 0.008	26.47 ± 0.410	0.094 ± 0.0076	0.917 ± 0.0956	17.8 ± 4.32
8,576	3.36 ± 0.046	0.000703 ± 0.00008	0.672 ± 0.015	9.346 ± 0.186	0.132 ± 0.0196	0.976 ± 0.108	3.27 ± 0.474
12,864	13.5 ± 0.261	0.000112 ± 0.00001	0.818 ± 0.004	6320 ± 141.7	0.000189 ± 0.0001	0.675 ± 0.0523	13.79 ± 1.66
17,152	21.9 ± 0.204	0.00453 ± 0.00181	0.433 ± 0.038	530.4 ± 219	0.00075 ± 0.00137	0.895 ± 0.307	3450.± 873
21,440	22.7 ± 0.248	0.00307 ± 0.0012	0.487 ± 0.049	18.83 ± 4.968	0.00094 ± 0.00001	0.782 ± 0.0125	4990 ± 656
25,728	23.61 ± 0.479	0.00931 ± 0.00576	0.34 ± 0.065	457.6 ± 90.3	0.00118 ± 0.0004	0.852 ± 0.0887	3370 ± 2150
30,016	29.8 ± 0.499	0.00996 ± 0.00562	0.335 ± 0.062	227 ± 169	0.00135 ± 0.0002	0.852 ± 0.0667	2300 ± 825
32160	37.0 ± 0.823	0.0122 ± 0.0128	0.305 ± 0.107	1590 ± 163	0.00164 ± 0.0009	0.826 ± 0.104	2050 ± 216

Table S4. Fitting parameters for each charge loading for the operating conditions: CD = 50 mA·cm⁻², PR interval = 5.36 seconds

Charge loading ($C \cdot L^{-1}$)	Rs (Ω·cm²)	Q1 (S·s^n/cm²)	n1	R1 (Ω·cm²)	Q2 (S·s^n/cm²)	n2	R2 (Ω·cm²)
215	2.05 ± 0.26	0.0002 ± 0.00003	0.897 ± 0.034	32.3 ± 2.92	0.0109 ± 0.0020	0.288 ± 0.020	217 ± 76.9
3,216	5.14 ± 0.05	0.0001 ± 0.00006	0.786 ± 0.006	80.96 ± 0.98	0.0310 ± 0.0021	0.990 ± 0.080	56.7 ± 11.49
6,432	5.36 ± 0.06	0.00028 ± 0.00001	0.693 ± 0.006	103.7 ± 1.99	0.0174 ± 0.0015	1.000 ± 0.086	51.96 ± 7.69
9,648	4.05 ± 0.16	0.0002 ± 0.00002	0.825 ± 0.025	55.21 ± 4.30	0.0096 ± 0.0019	0.326 ± 0.018	255.1 ± 89
12,864	7.01 ± 0.08	0.00048 ± 0.00003	0.648 ± 0.009	60.8 ± 1.47	0.0145 ± 0.0010	1.000 ± 0.060	43.45 ± 4.14
16,080	7.58 ± 0.48	0.0194 ± 0.0003	0.222 ± 0.024	3190 ± 364	0.0001 ± 0.00002	0.940 ± 0.033	27.5 ± 1.91
22,512	9.13 ± 0.64	0.0199 ± 0.0003	0.204 ± 0.024	1200 ± 538	0.0001 ± 0.00004	1.000 ± 0.049	15.68 ± 1.51
25,728	17.66 ± 0.18	0.0045 ± 0.004	0.864 ± 0.090	22.42 ± 3.95	0.0005 ± 0.0001	0.689 ± 0.027	20.85 ± 0.80
28,944	19.93 ± 0.22	0.0004 ± 0.0009	0.687 ± 0.031	18.05 ± 0.65	0.069 ± 0.007	0.903 ± 0.110	16.23 ± 3.61
32,160	20.21 ± 0.20	0.0005 ± 0.0001	0.685 ± 0.028	22.82 ± 0.90	0.044 ± 0.004	0.899 ± 0.098	20.56 ± 3.64

Table S5. Fitting parameters for each charge loading for the operating conditions: CD = 50 mA·cm⁻², PR interval = 53.6 seconds

**Charge loading ($C \cdot L^{-1}$)**	**Rs (Ω·cm²)**	**Q1 ($S \cdot s^n/cm^2$)**	**n1**	**R1 (Ω·cm²)**	**Q2 (S·s^n/cm²)**	**n2**	**R2 (Ω·cm²)**
215	3.05 ± 0.05	0.0001 ± 0.00004	0.869 ± 0.025	6.047 ± 0.14	0.121 ± 0.00886	0.862 ± 0.0624	8.286 ± 1.06
4,288	11.1 ± 0.12	0.0003 ± 0.0001	0.779 ± 0.041	6.76 ± 0.293	0.149 ± 0.0269	0.799 ± 0.142	5.077 ± 1.28
8,576	14.59 ± 1.5	0.0003 ± 0.00004	0.846 ± 0.19	2.867 ± 1.16	0.209 ± 0.192	0.163 ± 0.212	28.31 ± 186
12,864	15.55 ± 21.8	0.08 ± 0.01	0.0599 ± 0.35	985.3 ± 311	0.00022 ± 0.00047	1.0 ± 0.324	1.913 ± 1.09
17,152	19.98 ± 0.28	0.8 ± 0.8	0.503 ± 1.06	8860 ± 114	0.00174 ± 0.00162	0.612 ± 0.122	4.803 ± 1.12
21,440	20.73 ± 0.73	0.017 ± 0.003	0.609 ± 0.29	4.048 ± 4.35	0.393 ± 0.511	0.281 ± 1.298	29400 ± 104
25,728	18.57 ± 2.15	0.0015 ± 0.0003	1.0 ± 0.24	2.219 ± 0.91	0.136 ± 0.114	0.136 ± 0.153	83.87 ± 99
30,016	17.67 ± 9.48	0.78 ± 0.117	0.0846 ± 0.23	63.96 ± 7.5	0.00019 ± 0.00036	1.0 ± 0.280	2.267 ± 1.11
32,160	20.66 ± 5.33	0.0001 ± 0.0001	1.0 ± 0.208	3.11 ± 1.08	0.116 ± 0.176	0.0957 ± 0.215	2930 ± 195

Table S6. Fitting parameters for each charge loading for the operating conditions: CD = 50 mA·cm⁻², PR interval = 536 seconds

Charge loading ($C \cdot L^{-1}$)	Rs ($\Omega \cdot cm^2$)	Q1 ($S \cdot s^n/cm^2$)	n1	R1 ($\Omega \cdot cm^2$)	Q2 (S·s^n/cm²)	n2	R2 ($\Omega \cdot cm^2$)
215	2.28 ± 0.04	0.00008 ± 0.0001	0.75 ± 0.008	721.1 ± 200	0.0003 ± 0.0001	1 ± 0.02	5790 ± 300
4,288	6.73 ± 0.07	0.213 ± 0.03	0.841 ± 0.1	3.326 ± 0.6	0.000688 ± 0.0003	0.793 ± 0.05	3.062 ± 0.2
8,576	11 ± 0.1	0.249 ± 0.07	0.751 ± 0.2	2.628 ± 1	0.00119 ± 0.0008	0.726 ± 0.09	3.029 ± 0.3
12,864	13.93 ± 0.2	0.203 ± 0.06	0.843 ± 0.2	2.918 ± 1	0.0027 ± 0.002	0.625 ± 0.1	3.635 ± 0.5
17,152	13.63 ± 4	0.000486 ± 0.001	1 ± 0.3	1.201 ± 0.7	0.192 ± 0.4	0.0903 ± 0.3	217.8 ± 200
21,440	17.73 ± 0.3	0.417 ± 0.3	0.65 ± 0.6	2.709 ± 4	0.00354 ± 0.004	0.561 ± 0.1	3.789 ± 1
25,728	20.68 ± 0.3	0.266 ± 0.1	0.839 ± 0.4	2.108 ± 1	0.00273 ± 0.003	0.573 ± 0.1	4.095 ± 0.8
30,016	20.51 ± 10	0.0933 ± 0.2	0.0847 ± 0.4	17.75 ± 10	0.000278 ± 0.0009	1 ± 0.5	1.357 ± 1
32,160	19.97 ± 20	0.121 ± 0.6	0.0687 ± 0.7	38.33 ± 10	0.00322 ± 0.01	0.667 ± 0.7	1.62 ± 4

Table S7. Fitting parameters for each charge loading for the operating conditions: CD = 100 mA·cm⁻², no PR

Charge loading (C·L⁻¹)	Rs (Ω·cm²)	Q1 (S·s^n/cm²)	n1	R1 (Ω·cm²)	Q2 (S·s^n/cm²)	n2	R2 (Ω·cm²)
215	3.34 ± 0.03	0.00013 ± 0.00001	0.817 ± 0.01	74.34 ± 0.89	0.06690 ± 0.0007	0.968 ± 0.148	31.96 ± 13.69
4,288	3.40 ± 0.04	0.00017 ± 0.00001	0.759 ± 0.01	28.33 ± 0.38	0.04280 ± 0.0004	0.941 ± 0.074	14.09 ± 1.60
8,576	3.64 ± 0.05	0.00037 ± 0.00003	0.688 ± 0.01	19.56 ± 0.30	0.06710 ± 0.0007	0.984 ± 0.085	8.28 ± 1.02
12,864	3.79 ± 0.05	0.08720 ± 0.001	1.000 ± 0.11	3.34 ± 0.47	0.00059 ± 0.0007	0.660 ± 0.015	11.49 ± 0.23
17,152	4.48 ± 0.07	0.13400 ± 0.0031	0.980 ± 0.15	2.37 ± 0.47	0.00112 ± 0.0001	0.604 ± 0.019	9.52 ± 0.27
21,440	5.97 ± 0.10	0.00107 ± 0.0003	0.614 ± 0.05	7.50 ± 1.43	0.21300 ± 0.822	0.422 ± 0.375	5.74 ± 7.04
25,728	12.00 ± 0.25	0.00102 ± 0.0003	0.538 ± 0.04	26.45 ± 4.41	0.00016 ± 0.0004	0.886 ± 0.007	6320. ± 183.50
30,016	23.32 ± 0.25	0.00102 ± 0.0003	0.545 ± 0.04	32.54 ± 4.41	0.00018 ± 0.0003	0.886 ± 0.007	7340 ± 120
32,160	23.20 ± 0.25	0.00085 ± 0.0003	0.566 ± 0.04	17.76 ± 2.49	0.00023 ± 0.00003	0.860 ± 0.006	7920± 333.3

Table S8. Fitting parameters for each charge loading for the operating conditions: CD = 100 mA·cm⁻², PR interval = 5.36 seconds

Charge loading ($C \cdot L^{-1}$)	Rs (Ω·cm²)	Q1 (S·s^n/cm²)	n1	R1 (Ω·cm²)	Q2 (S·s^n/cm²)	n2	R2 (Ω·cm²)
215	2.62 ± 0.03	0.0006 ± 0.00	0.82 ± 0.01	79.69 ± 1.37	0.04 ± 0.00	0.76 ± 0.12	95.68 ± 56.62
4,288	3.40 ± 0.04	0.0005 ± 0.00	0.78 ± 0.01	20.08 ± 0.32	0.07 ± 0.01	0.77 ± 0.07	24.52 ± 5.34
8,576	3.73 ± 0.05	0.0004 ± 0.00	0.72 ± 0.01	17.75 ± 0.34	0.06 ± 0.00	0.79 ± 0.07	19.87 ± 3.27
12,864	4.11 ± 0.05	0.0004 ± 0.00	0.66 ± 0.01	33.87 ± 0.71	0.04 ± 0.00	0.87 ± 0.07	30.38 ± 4.74
17,152	4.67 ± 0.06	0.004 ± 0.00	0.63 ± 0.01	38.05 ± 0.89	0.03 ± 0.00	0.91 ± 0.07	33.27 ± 4.14
21,440	5.32 ± 0.07	0.0003 ± 0.00	0.60 ± 0.01	36.79 ± 0.99	0.03 ± 0.00	0.94 ± 0.06	32.74 ± 3.60
25,728	6.10 ± 0.10	0.0003 ± 0.00	0.56 ± 0.01	27.65 ± 0.91	0.03 ± 0.00	0.87 ± 0.07	30.16 ± 4.00
30,016	7.21 ± 0.11	0.0003 ± 0.00	0.62 ± 0.03	9.76 ± 0.45	0.07 ± 0.01	0.73 ± 0.08	11.46 ± 1.94
32,160	11.96 ± 0.41	0.0004 ± 0.00	0.73 ± 0.11	5.90 ± 1.92	0.19 ± 0.08	0.30 ± 0.34	22200 ± 751

Table S9. Fitting parameters for each charge loading for the operating conditions: CD = 100 mA·cm^{-2}, PR interval = 53.6 seconds

Charge loading (C·L^{-1})	Rs (Ω·cm²)	Q1 (S·s^n/cm²)	n1	R1 (Ω·cm²)	Q2 (S·s^n/cm²)	n2	R2 (Ω·cm²)
215	2.13 ± 0.02	0.0006 ± 0.0001	0.87 ± 0.01	7.69 ± 0.13	0.036 ± 0.001	1.00 ± 0.02	22.8 ± 1.1
4,288	2.67 ± 0.02	0.0005 ± 0.0001	0.89 ± 0.02	4.61 ± 0.10	0.082 ± 0.003	0.85 ± 0.03	13.7 ± 1.1
8,576	3.65 ± 0.03	0.129 ± 0.014	0.69 ± 0.07	4.87 ± 0.64	0.0004 ± 0.0001	0.90 ± 0.03	3.22 ± 0.14
12,864	4.61 ± 0.04	0.0004 ± 0.0002	0.87 ± 0.05	2.22 ± 0.11	0.15 ± 0.02	0.80 ± 0.08	3.30 ± 0.39
17,152	5.37 ± 0.06	0.0003 ± 0.0001	0.84 ± 0.05	2.77 ± 0.16	0.11 ± 0.02	0.71 ± 0.08	3.70 ± 0.49
21,440	6.76 ± 0.08	0.0025 ± 0.0009	0.64 ± 0.05	4.47 ± 0.31	0.024 ± 0.001	0.86 ± 0.03	39.9 ± 2.6
25,728	7.99 ± 0.13	0.0042 ± 0.0025	0.57 ± 0.08	3.26 ± 0.43	0.17 ± 0.03	0.75 ± 0.15	4.09 ± 1.09
32,160	12.8 ± 0.03	0.129 ± 0.014	0.69 ± 0.07	4.87 ± 0.63	0.0004 ± 0.0001	0.90 ± 0.03	5.22 ± 0.14

Table S10. Fitting parameters for each charge loading for the operating conditions: CD = 100 mA·cm⁻², PR interval = 536 seconds

Charge loading ($C \cdot L^{-1}$)	Rs (Ω·cm²)	Q1 (S·s^n/cm²)	n1	R1 (Ω·cm²)	Q2 (S·s^n/cm²)	n2	R2 (Ω·cm²)
215	3.13 ± 0.03	0.0005 ± 0.00	0.72 ± 0.01	14.59 ± 0.25	0.06 ± 0.00	0.99 ± 0.05	17.82 ± 1.60
4,288	3.55 ± 0.03	0.0004 ± 0.00	0.82 ± 0.03	3.88 ± 0.10	0.14 ± 0.01	0.90 ± 0.05	6.86 ± 0.67
8,576	3.48 ± 0.03	0.0003 ± 0.00	0.85 ± 0.03	2.77 ± 0.09	0.18 ± 0.01	0.87 ± 0.06	4.55 ± 0.51
12,864	3.87 ± 0.04	0.0005 ± 0.00	0.82 ± 0.05	1.75 ± 0.09	0.32 ± 0.05	0.81 ± 0.11	2.00 ± 0.35
17,152	4.31 ± 0.04	0.0004 ± 0.00	0.87 ± 0.06	1.60 ± 0.09	0.38 ± 0.07	0.79 ± 0.13	1.74 ± 0.38
21,440	7.23 ± 0.08	0.0004 ± 0.00	0.71 ± 0.13	1.41 ± 0.23	0.40 ± 0.17	0.75 ± 0.29	1.16 ± 0.52
25,728	12.11 ± 0.53	0.51 ± 0.69	0.98 ± 0.98	1.05 ± 1.71	0.00 ± 0.00	0.38 ± 0.03	20.95 ± 1.65
30,016	13.82 ± 0.40	0.00 ± 0.00	0.70 ± 0.03	41.58 ± 2.52	0.00 ± 0.00	0.55 ± 0.02	577.40 ± 57.28
32,160	15.81 ± 0.39	0.00 ± 0.00	0.72 ± 0.02	67.16 ± 4.35	0.00 ± 0.00	0.57 ± 0.03	463.80 ± 32.76

Table S11. Fitting parameters for each charge loading for the operating conditions: CD = 200 mA·cm⁻², no PR

Charge loading (C·L^{-1})	Rs (Ω·cm²)	Q1 (S·s^n/cm²)	n1	R1 (Ω·cm²)	Q2 (S·s^n/cm²)	n2	R2 (Ω·cm²)
215	1.98 ± 0.02	0.00021 ± 0.00001	0.782 ± 0.01	21.50 ± 0.2	0.08 ± 0.01	1.00 ± 0.08	8.91 ± 1.14
4,288	2.07 ± 0.03	0.00031 ± 0.00002	0.750 ± 0.01	15.60 ± 0.2	0.11 ± 0.01	0.96 ± 0.10	5.42 ± 0.80
8,576	1.99 ± 0.03	0.00041 ± 0.00004	0.726 ± 0.01	11.50 ± 0.2	0.16 ± 0.02	0.93 ± 0.11	3.72 ± 0.63
12,864	2.00 ± 0.03	0.16100 ± 0.02440	0.873 ± 0.12	3.77 ± 0.7	0.00 ± 0.00	0.70 ± 0.01	10.70 ± 0.20
17,152	2.06 ± 0.03	0.00073 ± 0.00008	0.679 ± 0.01	9.06 ± 0.2	0.17 ± 0.03	0.86 ± 0.12	3.42 ± 0.62
21,440	2.11 ± 0.03	0.00080 ± 0.00009	0.676 ± 0.01	8.60 ± 0.2	0.17 ± 0.03	0.78 ± 0.12	3.94 ± 0.81
25,728	2.20 ± 0.03	0.00087 ± 0.00010	0.676 ± 0.01	7.72 ± 0.2	0.19 ± 0.03	0.81 ± 0.12	3.13 ± 0.63
30,016	2.27 ± 0.03	0.15900 ± 0.02120	0.820 ± 0.11	4.02 ± 0.7	0.00 ± 0.00	0.66 ± 0.01	7.82 ± 0.20
32,160	2.49 ± 0.03	0.00122 ± 0.00016	0.673 ± 0.02	6.46 ± 0.2	0.23 ± 0.03	0.80 ± 0.12	3.55 ± 0.79

Table S12. Fitting parameters for each charge loading for the operating conditions: CD = 200 mA·cm^{-2}, PR interval = 5.36 seconds

Charge loading (C·L^{-1})	Rs (Ω·cm²)	Q1 (S·s^n/cm²)	n1	R1 (Ω·cm²)	Q2 (S·s^n/cm²)	n2	R2 (Ω·cm²)
215	3.45 ± 0.04	4.66E-5 ± 1.71E-6	0.874 ± 0.005	159.9 ± 1.8	2.96E-2 ± 2.83E-3	0.918 ± 0.137	107.2 ± 61.6
4,288	3.36 ± 0.03	7.50E-5 ± 2.97E-6	0.871 ± 0.005	101.2 ± 1.1	3.84E-2 ± 3.47E-3	1.000 ± 0.112	50.24 ± 15.2
8,576	3.41 ± 0.03	9.89E-5 ± 4.14E-6	0.837 ± 0.005	89.17 ± 1.0	3.53E-2 ± 2.99E-3	1.000 ± 0.099	44.47 ± 10.3
12,864	3.32 ± 0.03	1.15E-4 ± 5.08E-6	0.826 ± 0.006	78.4 ± 1.0	2.80E-2 ± 2.14E-3	0.927 ± 0.085	49.72 ± 9.6
17,152	3.45 ± 0.03	1.48E-4 ± 7.67E-6	0.818 ± 0.007	53.94 ± 0.88	2.66E-2 ± 1.92E-3	0.854 ± 0.073	43.46 ± 6.8
21,440	3.52 ± 0.04	1.80E-4 ± 9.45E-6	0.796 ± 0.007	53.09 ± 0.91	3.32E-2 ± 2.54E-3	0.841 ± 0.084	42.6 ± 8.6
25,728	3.77 ± 0.04	2.26E-4 ± 1.61E-5	0.771 ± 0.009	28.98 ± 0.49	3.79E-2 ± 2.47E-3	0.849 ± 0.061	28.21 ± 3.6
32,160	4.17 ± 0.05	6.06E-2 ± 4.39E-3	0.811 ± 0.062	16.76 ± 2.23	2.47E-4 ± 2.50E-5	0.765 ± 0.012	16.17 ± 0.30

Table S13. Fitting parameters for each charge loading for the operating conditions: CD = 200 mA·cm⁻², PR interval = 53.6 seconds

**Charge loading ($C \cdot L^{-1}$)**	**Rs (Ω·cm²)**	**Q1 (S·s^n/cm²)**	**n1**	**R1 (Ω·cm²)**	**Q2 (S·s^n/cm²)**	**n2**	**R2 (Ω·cm²)**
215	1.98 ± 0.02	0.00021 ± 0.00001	0.782 ± 0.01	21.50 ± 0.2	0.08 ± 0.01	1.00 ± 0.08	8.91 ± 1.14
4,288	2.07 ± 0.03	0.00031 ± 0.00002	0.750 ± 0.01	15.60 ± 0.2	0.11 ± 0.01	0.96 ± 0.10	5.42 ± 0.80
8,576	1.99 ± 0.03	0.00041 ± 0.00004	0.726 ± 0.01	11.50 ± 0.2	0.16 ± 0.02	0.93 ± 0.11	3.72 ± 0.63
12,864	2.00 ± 0.03	0.16100 ± 0.02440	0.873 ± 0.12	3.77 ± 0.7	0.00 ± 0.00	0.70 ± 0.01	10.70 ± 0.20
17,152	2.06 ± 0.03	0.00073 ± 0.00008	0.679 ± 0.01	9.06 ± 0.2	0.17 ± 0.03	0.86 ± 0.12	3.42 ± 0.62
21,440	2.11 ± 0.03	0.00080 ± 0.00009	0.676 ± 0.01	8.60 ± 0.2	0.17 ± 0.03	0.78 ± 0.12	3.94 ± 0.81
25,728	2.20 ± 0.03	0.00087 ± 0.00010	0.676 ± 0.01	7.72 ± 0.2	0.19 ± 0.03	0.81 ± 0.12	3.13 ± 0.63
30,016	2.27 ± 0.03	0.15900 ± 0.02120	0.820 ± 0.11	4.02 ± 0.7	0.00 ± 0.00	0.66 ± 0.01	7.82 ± 0.20
32,160	2.49 ± 0.03	0.00122 ± 0.00016	0.673 ± 0.02	6.46 ± 0.2	0.23 ± 0.03	0.80 ± 0.12	3.55 ± 0.79

Table S14. Fitting parameters for each charge loading for the operating conditions: CD = 200 mA·cm⁻², PR interval = 536 seconds

Charge loading ($C \cdot L^{-1}$)	Rs (Ω·cm²)	Q1 (S·s^n/cm²)	n1	R1 (Ω·cm²)	Q2 (S·s^n/cm²)	n2	R2 (Ω·cm²)
215	2.616 ± 0.0215	0.000741 ± 0.0000603	0.839 ± 0.0118	9.956 ± 0.174	0.0669 ± 0.00761	0.940 ± 0.049	14.53 ± 1.701
4,288	2.530 ± 0.0192	0.000707 ± 0.0000498	0.894 ± 0.0108	10.73 ± 0.169	0.0428 ± 0.00404	0.974 ± 0.064	11.71 ± 1.899
8,576	2.584 ± 0.0193	0.000914 ± 0.0000696	0.892 ± 0.0120	9.109 ± 0.159	0.0671 ± 0.00716	0.967 ± 0.069	9.487 ± 1.586
12,864	2.552 ± 0.0199	0.00132 ± 0.000131	0.850 ± 0.0154	6.228 ± 0.140	0.00059 ± 0.00007	0.901 ± 0.071	7.605 ± 1.298
17,152	2.741 ± 0.0211	0.00139 ± 0.000139	0.849 ± 0.0156	6.462 ± 0.143	0.00112 ± 0.00017	0.928 ± 0.070	7.931 ± 1.348
21,440	2.934 ± 0.0234	0.00175 ± 0.000207	0.814 ± 0.0182	5.536 ± 0.139	0.213 ± 0.00814	0.932 ± 0.067	6.886 ± 0.990
25,728	2.930 ± 0.0246	0.00234 ± 0.000326	0.771 ± 0.0210	4.657 ± 0.138	0.000165 ± 0.0002	0.917 ± 0.068	5.984 ± 0.836
30,016	2.990 ± 0.0224	0.000368 ± 0.0000135	0.903 ± 0.0063	66.55 ± 1.791	0.00016 ± 0.00002	1.000 ± 0.305	8.036 ± 3.526
32,160	2.616 ± 0.0215	0.000741 ± 0.0000603	0.839 ± 0.0118	9.956 ± 0.174	0.00022 ± 0.00003	0.940 ± 0.049	14.53 ± 1.701

Table S15. Paired sampled t-test parameters for hypothesis testing

Current Density ($mA \cdot cm^{-2}$)	PR interval (s)	Mean Difference (mV)	Standard mean error (mV)	t-Statistic	Degrees of freedom	p-Value	Significant ($p < 0.05$)
50	No PR	116.22	92.48	1.25	8	0.245	No
50	5.36	139.78	86.96	1.61	9	0.142	No
50	53.6	257.49	120.47	2.14	8	0.065	No
50	536	70.09	33.26	2.11	9	0.064	No
100	No PR	23.42	56.23	1.25	8	0.247	No
100	5.36	-14.06	64.12	-0.69	9	0.509	No
100	53.6	-83.72	125.99	-2.10	9	0.064	No
100	536	-66.11	121.18	-1.72	9	0.118	No
200	No PR	5.04	6.95	2.17	8	0.061	No
200	5.36	-5.15	12.37	-1.26	8	0.246	No
200	53.6	-3.546	8.47	-1.25	8	0.245	No
200	536	-0.539	12.86	-0.19	7	0.908	No

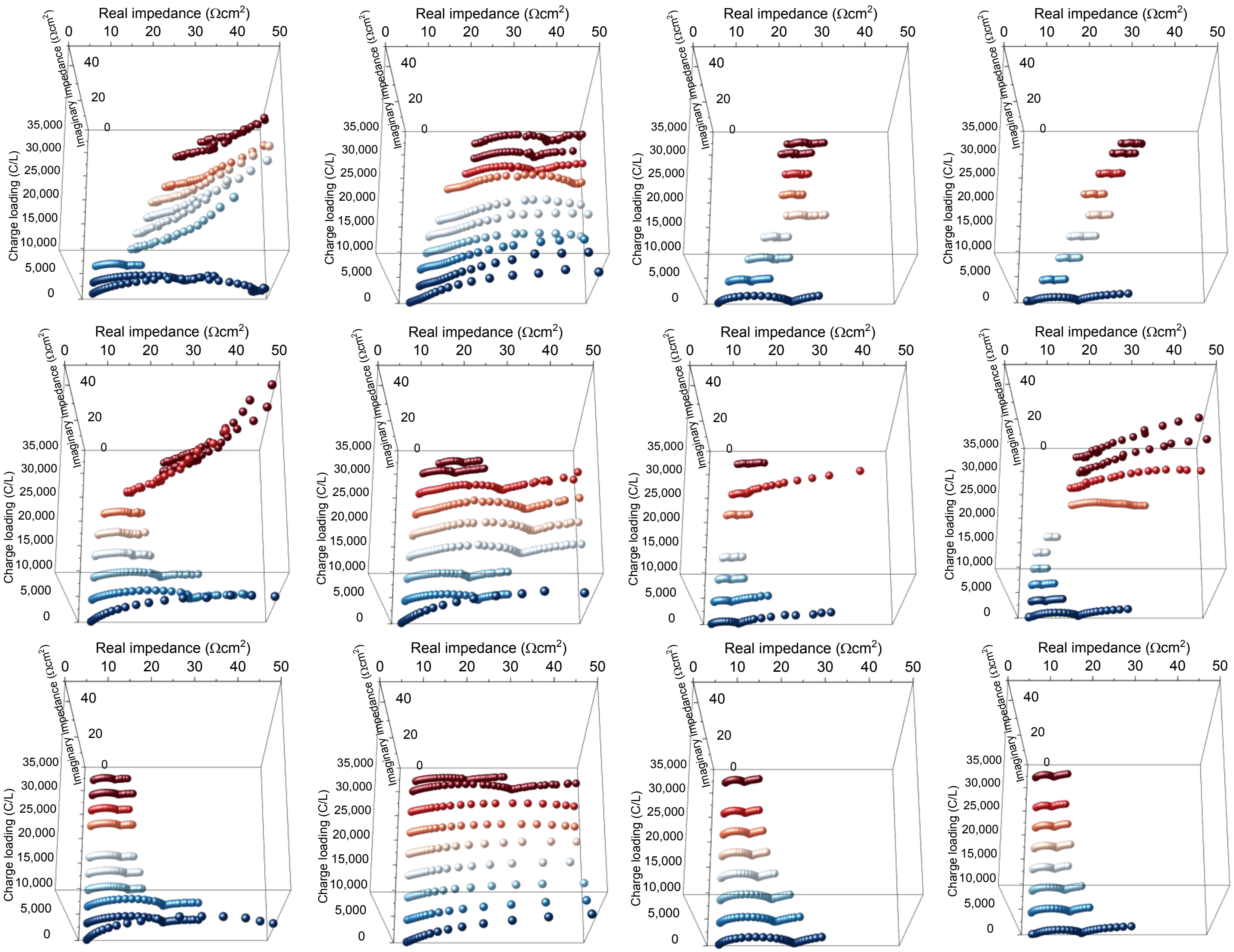

Figure S4. Three-dimentional Nyquist plots with charge loading for CD = 50 mA·cm^{-2} with (a) no PR, (b) PR interval = 5.36 s, (c) PR interval = 53.6 seconds, (d) PR interval = 536 s, CD = 100 mA·cm^{-2} with (e) no PR, (f) PR interval = 5.36 s, (g) PR interval = 53.6 s, (h) PR interval = 536 s, CD = 200 mA·cm^{-2} with (i) no PR, (j) PR interval = 5.36 s, (k) PR interval = 53.6 s, (l) PR interval = 536 s.

Section S10: Surface characterization of (electro)deposits and flocs

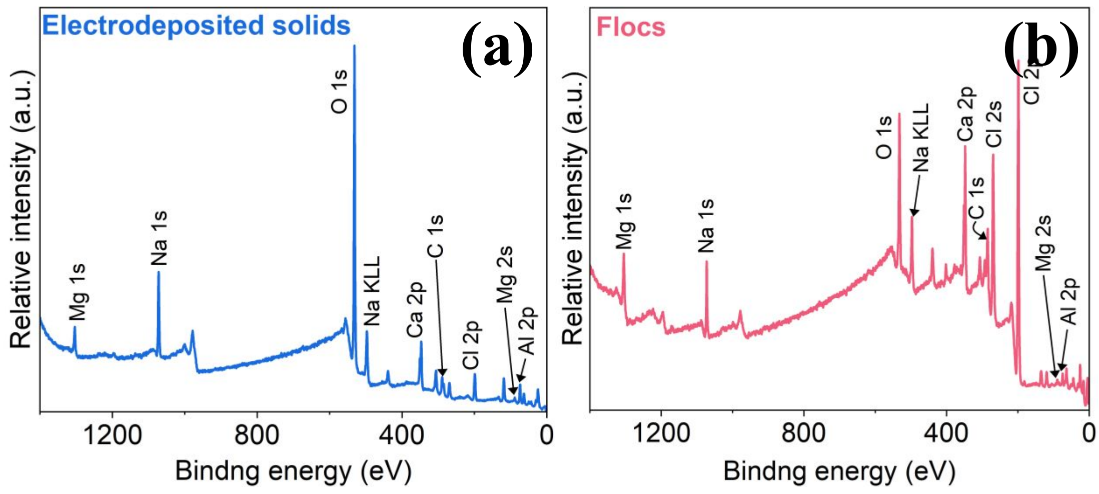

Figure S5. XPS survey scans for (a) scraped electrodeposited solids and (b) aluminum flocs, (c) aluminum flocs in synthetic NaCl solution, revealed similar elemental compositions, including sodium (Na), chlorine (Cl), calcium (Ca), magnesium (Mg), aluminum (Al), and carbon (C). The detection of sodium and chlorine is consistent with the hypersaline nature of the produced water. The presence of calcium and magnesium in the flocs suggests that these elements were dislodged from the electrode surface during electrocoagulation.

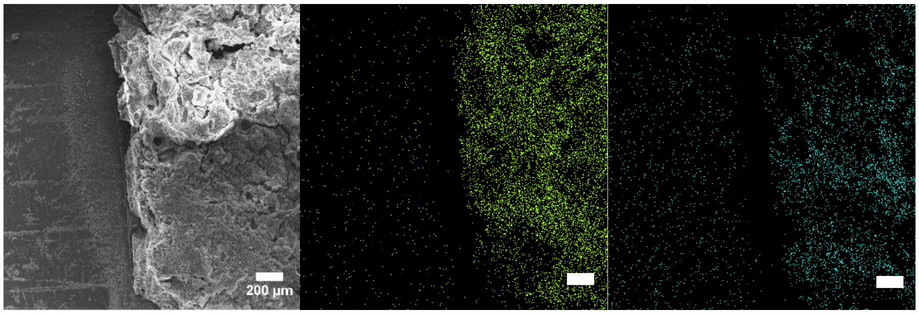

Figure S6. (a) Electron micrograph of virgin cathode, (b) EDS map showing calcium, (c) EDS map showing magnesium.

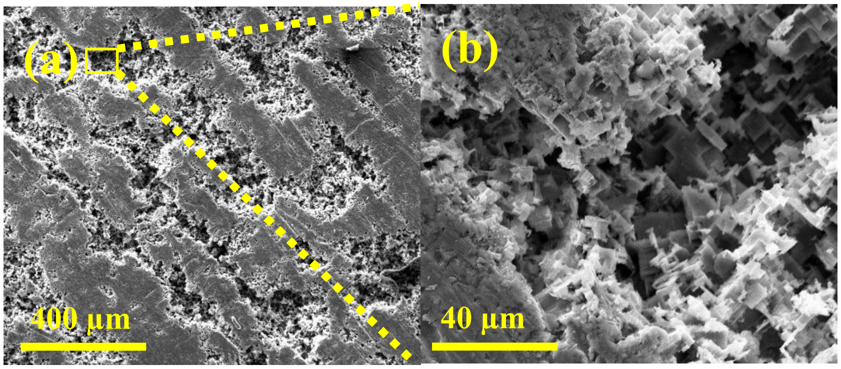

Figure S7. (a) Pitting on the anode, (b) magnified image of the a pit clearly showing crystallographic nature.

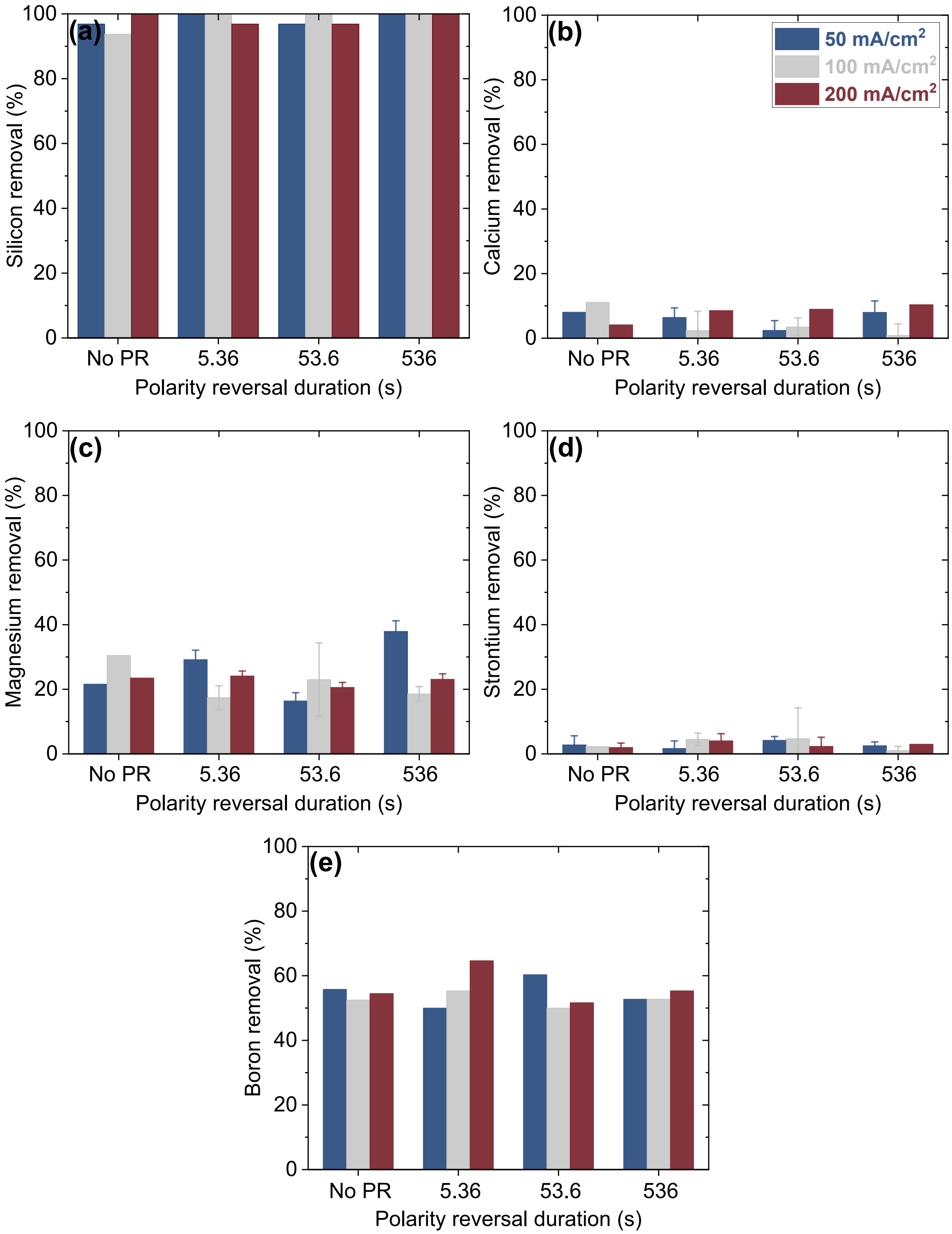

Figure S8. Removal (in %) of (a) silicon, (b) calcium, (c) magnesium, (d) strontium and (e) boron from produced water at tested CDs and PR intervals.

Both the suspended flocs and the electrodeposits were characterized using EDS and XPS. Notably, EDS of the flocs revealed the presence of calcium and magnesium (**SI Figure S10**), a finding that contrasts with our previous work, which attributed hardness removal solely to cathodic electrodeposition. To investigate this discrepancy, XPS survey spectra for the flocs was obtained which exhibited similar elemental compositions to the electrodeposited solids, with prominent signals for aluminum, calcium, magnesium, carbon, and oxygen (**SI Figure S5b**). The calcium and magnesium present in the electrodeposits likely precipitated under conditions of localized supersaturation in the high-pH environment near the cathode. Importantly, high-resolution XPS scans identified the dominant crystalline phases as calcium carbonate ($CaCO_3$) and magnesium hydroxide [$Mg(OH)_2$], both of which are thermodynamically favored only under alkaline conditions[21]. Given that the bulk solution pH was insufficient to support their supersaturation and nucleation, we conclude that these minerals could not have formed in the bulk electrolyte. Instead, their presence in the flocs suggests mechanical dislodgement from the cathode surface, facilitated by vigorous hydrogen evolution. This mechanism is supported by our own observations, where higher CDs were shown to intensify hydrogen bubbling, promoting not only the mitigation of cathodic fouling but also the detachment of loosely bound precipitates (compare **SI Figures S2c, d, g,** and **h** to **SI Figure S2k-l**)[9]. The aluminum in the flocs is consistent with the presence of amorphous aluminum hydroxide precipitates. In contrast, the aluminum detected in the electrodeposits is believed to originate from aluminum hydroxides that nucleated and precipitated heterogeneously onto growing calcium and magnesium crystals, as discussed in the earlier section.

The XPS survey spectra for aluminum flocs identified similar elemental compositions to the foulants, with prominent signals for aluminum, calcium, magnesium, sodium, chloride, carbon, and oxygen (**SI Figure S5b**). Aluminum in the flocs is consistent with the presence of amorphous aluminum hydroxide precipitates, while sodium and chloride were believed to originate from the drying process of the flocs.

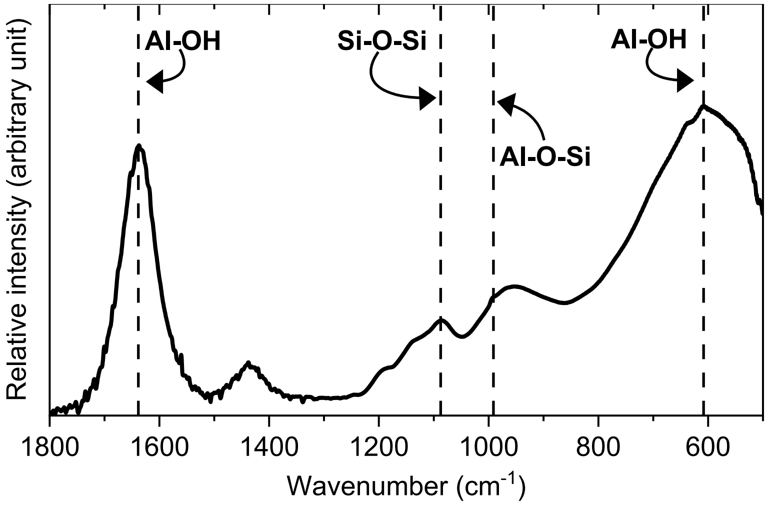

Figure S9. IR spectra of flocs revealing bonding between silicon and aluminum.

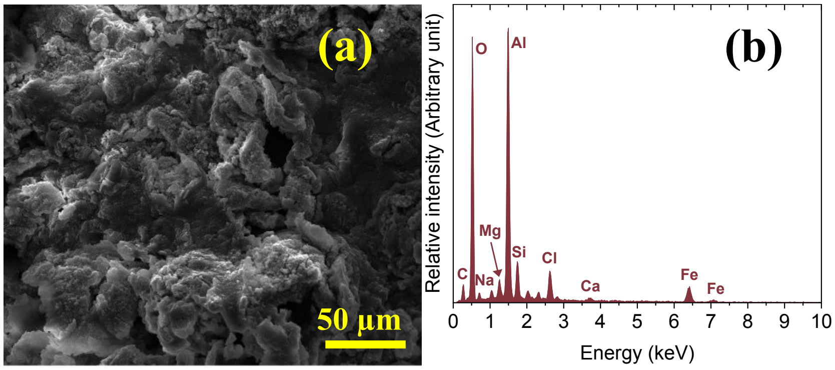

Figure S10. (a) Electron micrograph of floc, (b) corresponding EDS spectrum

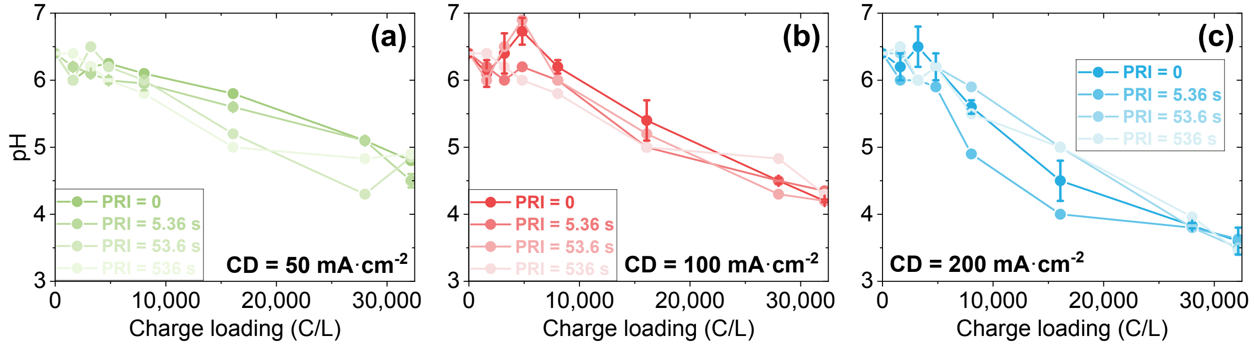

Figure S11. pH variations with charge loading (C/L) for (a) 50 $mA \cdot cm^{-2}$, (a) 100 $mA \cdot cm^{-2}$, (c) 200 $mA \cdot cm^{-2}$. PRI represents polarity reversal interval.

Acknowledgements. SEM–EDS was conducted at the Texas A&M University Microscopy and Imaging Center (RRID: SCR_022128). Himani Deshpande 3D-printed the reactor lids and Akshat Verma performed ICP analyses. Partial funding from the National Science Foundation (CBET-2211035) is appreciated.

References

(1) APHA. *Standard Methods for the Examination of Water and Wastewater*; American Public Health Association, Washington, DC, USA., **2005**.

(2) Li, H.; Dzombak, D.; Vidic, R. Electrochemical impedance spectroscopy (EIS) based characterization of mineral deposition from precipitation reactions. *Industrial & Engineering Chemistry Research* **2012**, *51* (7), 2821-2829, DOI: https://doi.org/10.1021/ie201802n.

(3) Logan, B. E.; Zikmund, E.; Yang, W.; Rossi, R.; Kim, K.-Y.; Saikaly, P. E.; Zhang, F. Impact of Ohmic Resistance on Measured Electrode Potentials and Maximum Power Production in Microbial Fuel Cells. *Environmental Science & Technology* **2018**, *52* (15), 8977-8985, DOI: https://doi.org/10.1021/acs.est.8b02055.

(4) Abada, B.; Joag, S.; Sharma, R.; Chellam, S. Hypersaline produced water clarification by dissolved air flotation and sedimentation with ultrashort residence times. *Water Research* **2022**, 119241, DOI: https://doi.org/10.1016/j.watres.2022.119241.

(5) Major, G. H.; Fairley, N.; Sherwood, P. M. A.; Linford, M. R.; Terry, J.; Fernandez, V.; Artyushkova, K. Practical guide for curve fitting in x-ray photoelectron spectroscopy. *Journal of Vacuum Science & Technology A* **2020**, *38* (6), 061203, DOI: https://doi.org/10.1116/6.0000377.

(6) Kelly, R. G.; Scully, J. R.; Shoesmith, D.; Buchheit, R. G. *Electrochemical techniques in corrosion science and engineering*; CRC Press, ISBN: 0429221622, **2002**.

(7) McCafferty, E. *Introduction to corrosion science*; Springer Science & Business Media, ISBN: 1441904549, **2010**.

(8) Ghanbari, E.; Saatchi, A.; Lei, X.; Macdonald, D. D. Studies on pitting corrosion of Al-Cu-Li alloys part II: Breakdown potential and pit initiation. *Materials* **2019**, *12* (11), 1786, DOI: https://doi.org/10.3390/ma12111786.

(9) Chow, H.; Pham, A. L.-T. Mitigating electrode fouling in electrocoagulation by means of polarity reversal: The effects of electrode type, current density, and polarity reversal frequency. *Water Research* **2021**, *197*, 117074, DOI: https://doi.org/10.1016/j.watres.2021.117074.

(10) Jang, G. G.; Castillo, C.; Dutta, S.; Su, Y.-F.; Jun, J.; Keum, J. K.; Kim, K.; Chellam, S.; Tsouris, C. Performance restoration of iron electrodes by polarity reversal after long-term surface fouling in wastewater electrocoagulation. *ACS ES&T Water* **2024**, *4* (6), 2390-2402, DOI: https://doi.org/10.1021/acsestwater.3c00741.

(11) Chow, H.; Ingelsson, M.; Roberts, E. P. L.; Pham, A. L.-T. How does periodic polarity reversal affect the Faradaic efficiency and electrode fouling during iron electrocoagulation? *Water Research* **2021**, *203*, 117497, DOI: https://doi.org/10.1016/j.watres.2021.117497.

(12) Fuladpanjeh-Hojaghan, B.; Shah, R. S.; Roberts, E. P. L.; Trifkovic, M. Effect of polarity reversal on floc formation and rheological properties of a sludge formed by the electrocoagulation process. *Water Research* **2023**, *242*, DOI: https://doi.org/10.1016/j.watres.2023.120201.

(13) Müller, S.; Behrends, T.; van Genuchten, C. M. Sustaining efficient production of aqueous iron during repeated operation of Fe(0)-electrocoagulation. *Water Research* **2019**, *155*, 455-464, DOI: https://doi.org/10.1016/j.watres.2018.11.060.

(14) Liu, Y.-H.; Bootwala, Y. Z.; Jang, G. G.; Keum, J. K.; Khor, C. M.; Hoek, E. M. V.; Jassby, D.; Tsouris, C.; Mothersbaugh, J.; Hatzell, M. C. Electroprecipitation mechanism enabling silica and hardness removal through aluminum-based electrocoagulation. *ACS ES&T Engineering* **2022**, *2*, 1200-1210, DOI: https://doi.org/10.1021/acsestengg.1c00433.

(15) Joag, S.; Kiesewetter, J.; Chellam, S. Electrode corrosion, pH, and dissolved oxygen dynamics, and hardness/silicon removal during aluminum electrocoagulation of hypersaline produced water. *ACS ES&T Engineering* **2024**, *5* (1), 86-102, DOI: https://doi.org/10.1021/acsestengg.4c00459.

(16) Ellerbrock, R.; Stein, M.; Schaller, J. Comparing amorphous silica, short-range-ordered silicates and silicic acid species by FTIR. *Scientific Reports* **2022**, *12* (1), 11708, DOI: 10.1038/s41598-022-15882-4.

(17) Chorghe, D.; Sari, M. A.; Chellam, S. Boron removal from hydraulic fracturing wastewater by aluminum and iron coagulation: Mechanisms and limitations. *Water Research* **2017**, *126*, 481-487, DOI: https://doi.org/10.1016/j.watres.2017.09.057.

(18) Chen, M.; Dollar, O.; Shafer-Peltier, K.; Randtke, S.; Waseem, S.; Peltier, E. Boron removal by electrocoagulation: Removal mechanism, adsorption models and factors influencing removal. *Water research* **2020**, *170*, 115362, DOI: https://doi.org/10.1016/j.watres.2019.115362.

(19) Sari, M. A.; Chellam, S. Mechanisms of boron removal from hydraulic fracturing wastewater by aluminum electrocoagulation. *Journal of Colloid and Interface Science* **2015**, *458*, 103-111, DOI: https://doi.org/10.1016/j.jcis.2015.07.035.

(20) O'Donnell, A. J.; Lytle, D. A.; Harmon, S.; Vu, K.; Chait, H.; Dionysiou, D. D. Removal of strontium from drinking water by conventional treatment and lime softening in bench-scale studies. *Water Research* **2016**, *103*, 319-333, DOI: https://doi.org/10.1016/j.watres.2016.06.036.

(21) James, M.; Mitch, W. A. Electrochemical generation of hydroxide for precipitative water softening. *ACS ES&T Water* **2022**, *2* (12), 2677-2685, DOI: https://doi.org/10.1021/acsestwater.2c00451.
